# Tactile Low Frequency Vibration in Dementia Management: A Scoping Review

**DOI:** 10.3389/fpsyg.2022.854794

**Published:** 2022-06-17

**Authors:** Elsa A. Campbell, Jiří Kantor, Lucia Kantorová, Zuzana Svobodová, Thomas Wosch

**Affiliations:** ^1^Caritas Association Ettlingen, Ettlingen, Germany; ^2^VIBRAC Skille-Lehikoinen Centre for Vibroacoustic Therapy and Research, Eino Roiha Foundation, Jyväskylä, Finland; ^3^Center of Evidence-Based Education and Arts Therapies: A JBI Affiliated Group, Institute of Special Education Sciences, Faculty of Education, Palacky University, Olomouc, Czechia; ^4^Faculty of Medicine, Czech National Centre for Evidence-Based Healthcare and Knowledge Translation (Cochrane Czech Republic, Czech EBHC: JBI Centre of Excellence, Masaryk University GRADE Centre), Institute of Biostatistics and Analyses, Masaryk University, Brno, Czechia; ^5^Faculty of Health Sciences, Palacky University, Olomouc, Czechia; ^6^Institute for Applied Social Sciences, University of Applied Social Sciences, Würzburg-Schweinfurt, Germany

**Keywords:** low frequency vibration, dementia, vibroacoustic, whole body vibration, scoping review

## Abstract

The prevalence of dementia is increasing with the ever-growing population of older adults. Non-pharmacological, music-based interventions, including sensory stimulation, were reported by the Lancet Commission in 2020 to be the first-choice approach for managing the behavioural and psychological symptoms of dementia. Low frequency sinusoidal vibration interventions, related to music interventions through their core characteristics, may offer relief for these symptoms. Despite increasing attention on the effectiveness of auditory music interventions and music therapy for managing dementia, this has not included low frequency vibration. This scoping review, following the JBI methodology guidelines, was conducted to investigate participants’ responses to both sound and mechanical vibration, the characteristics of the delivered interventions, methodological challenges, and the specifics of the research experiments reported. An extensive search was conducted in BMC, CINAHL, Cochrane Central Register of Controlled Trials, EMBASE, ERIC, MEDLINE (OvidSP), Pedro, ProQuest Central, PsycINFO, Scopus, and Web of Science. Current Controlled Trials, Clinical Trials, and Google Scholar were also searched as well as a hand search in relevant journals. Studies on adults with all types of dementia, investigating tactile low frequency sound or mechanical vibration in any context were considered. Data from eight full-length studies (three RCTs, two quasi-experimental, two case reports, and one qualitative) were extracted using the data extraction table developed by the authors and were included in the analysis and critical appraisal. Issues in quality related to, for example, control groups and blinding. Few studies addressed participants’ subjective responses to the interventions. Reporting on the intervention characteristics was unclear. It appeared more frequent sessions led to better outcomes and home-based interventions potentially addressing the issue of access and feasibility. Future research should include neuroimaging to measure and confirm the hypothesised mechanism of cerebral coherence. Standardised reporting of intervention characteristics is also needed to ensure replicability of the experiments. Higher quality research is needed to investigate the impact and effect of low frequency vibration for the symptoms of dementia and compare outcomes in meta-syntheses.

## Introduction

The prevalence of dementia is set to rise with the increasing population of older adults (Livingston et al., 2020). Livingston and colleagues report that although pharmacological intervention is typically prescribed for managing the behavioural and psychological symptoms of dementia (BPSD), it should only be prescribed in severe cases and only when psychosocial intervention alone is insufficient. Medications for managing the symptoms of dementia however have reported side effects and are potentially harmful. Music-based interventions in dementia care have been receiving increasing attention in recent years and systematic reviews have shown improvements in agitation (Van der Steen et al., 2018), disruptive behaviour and anxiety, depression, cognitive functioning and quality of life (Zhang et al., 2017), as well as on physiological outcomes such as heart rate and blood pressure (Vasionytė and Madison, 2013).

There has been less attention on the vibrotactile aspects of music. Music is fundamentally sound and may be perceived in two forms of energy: sound and mechanical (Gunther, 2012). Since sound energy is primarily perceived through the ear because it lies within the human hearing range and mechanical energy is perceived primarily through touch since it is lower in frequency, music perception is a combination of these (Schneck and Berger, 2005). Humans can perceive sound waves through the ear between 20 and 20,000 Hz, but can detect frequencies below 20 Hz through tactile sensation. Sensitivity to tactile sensation increases from around 40 to about 250 Hz (Verrillo, 1992). Despite touch and body perception being important aspects of music therapy practice, for example kangaroo care for reducing anxiety (Kostilainen et al., 2021) or monochord for relaxation (Sandler et al., 2017), these aspects are not addressed in systematic reviews of music-based interventions or music therapy.

Two forms of tactile low frequency vibration are prevalent; low frequency sinusoidal sound vibration, otherwise referred to as, e.g., vibroacoustic therapy, physioacoustic therapy, resonant sensory stimulation (Ala-Ruona and Punkanen, 2017), and tactile low frequency mechanical vibration, such as whole body vibration (Cardinale and Wakeling, 2005; Oroszi et al., 2020; van Heuvelen et al., 2021). Vibroacoustic therapy, a receptive music therapy method (Grocke and Wigram, 2006) which combines sound vibration between 20 and 120 Hz, music listening, and a therapeutic relationship (Campbell, 2019), has rarely been discussed within the music therapy literature despite conforming to Bruscia's (1989) definition of music therapy, as delineated by Hooper (2001). Definition of music therapy Bruscia (1989), as delineated by Hooper (2001). It has also been applied as a standalone therapy or as part of a rehabilitation programme for managing chronic pain within specialised healthcare (Campbell et al., 2019). Whole body vibration delivers sinusoidal vibration between 15 and 60 Hz, displacements from <1 to 10 mm, and acceleration reaching 15 *g* through specially designed vertically or horizontally oscillating platforms (Cardinale and Wakeling, 2005; van Heuvelen et al., 2021). Although these interventions have until this point been discussed separately (vibroacoustic therapy mostly within the context of music interventions and whole body vibration as a method applied in sports rehabilitation), the underlying mechanism may be the same (Bartel and Mosabbir, 2021) and as such the mode of vibration perception may not necessarily be linked to its potential effectiveness. Monteiro et al. (2021), in a systematic review of different types of mechanical vibration for Alzheimer’s disease (AD), reported various methods are effective in relieving parameters of AD pathology such as neuronal circuit integrity deficits. The current scoping review discusses both sound and mechanical interventions which comprise a sinusoidal, low frequency stimulus; given the low frequency range, these are perceived both by the ear as a low humming and through tactile perception. Both interventions therefore utilise music as an integral part of the somatosensory stimulation, with the music being perceived in a tactile manner, i.e., as vibration. Given the Lancet Commission directive (Livingston et al., 2020) that psychosocial approaches—including sensory stimulation—be the first approach taken to manage (BPSD) in persons with dementia, the absence of sound and mechanical vibration from the literature highlights a gap in knowledge.

The scoping review method was chosen here rather than a systematic review since, according to Peters et al. (2020), scoping reviews are broader in scope, exploratory and descriptive, and can be used to identify and analyse gaps in knowledge and types of available evidence in the field. Furthermore, key characteristics related to a concept can be identified as well as how research is conducted in the field. This review aims to map the field of low frequency vibration for people with dementia, identify the types of evidence available, as well as explore the gaps in the literature, and investigate how the research has been conducted and reported. After a preliminary search in Epistemonikos, Cochrane Review, JBI Evidence Synthesis, Open Science Framework, and the International prospective register of systematic reviews (PROSPERO), we did not identify any systematic or scoping reviews on the topic.

## Materials and Methods

This review was guided by the following review questions:

- “What participant responses are reported in studies on tactile low frequency vibration and dementia and how have they been measured?”
- “What intervention characteristics are reported in studies on tactile low frequency vibration and dementia and how do these compare or differ across the approaches?,” and
- “What are the specifics of the research experiments in studies on tactile low frequency vibration and dementia?”

To address these questions, a scoping review method was applied and conducted in accordance with the JBI methodology for scoping reviews (Aromataris and Munn, 2020) and the extended Preferred Reporting Items for Systematic Reviews and Meta-Analyses (PRISMA) guidelines for scoping reviews (PRISMA-ScR; Munn et al., 2018; Tricco et al., 2018; Peters et al., 2020). The review was conducted according to an *a priori* published protocol (Campbell et al., 2021).

### Inclusion Criteria

The inclusion criteria were defined based on the Population—Concept—Context (PCC) components of the aforementioned review questions and are as follows:

- Participants: adults with all types of dementia, both genders, and having any level of cognitive/physical functioning. No age limit was set so as to include all studies reporting on any adults with dementia including types with younger adults such as Huntington’s disease. Studies with participants with comorbidities were included if dementia was the focus of the study.
- Concept: studies investigating the use of tactile low frequency vibration, e.g., Vibroacoustic therapy (VAT), with or without music listening, and with or without a therapeutic relationship, for managing dementia symptoms. Studies on whole body vibration (WBV) or interventions applying local mechanical vibration were also included. Multisensory environments in which vibration is a part and in which the participants’ responses to the low frequency vibration can be distinguished from their responses to the other stimuli were also included. Studies investigating electrical vibrations (e.g., transcranial magnetic stimulation or non-technology based low tones from singing bowls) were excluded.
- Context: no limitation was placed on the demographic context or study setting. Studies from all over the world conducted in residential care homes, hospitals, and private settings etc. were included. There was no limit on language given the title and abstract were in English. All types of studies were included in the search including quantitative, qualitative and mixed-methods, systematic and scoping reviews. The quantitative study designs included experimental, quasi-experimental, observational, case series/case studies, cross-sectional studies, and case reports from clinical practice. The qualitative studies were not limited by paradigm.

### Search Strategy

We conducted a comprehensive search of published and unpublished sources. These were conducted in EMBASE, CINAHL plus, Controlled Clinical Trials, BMC (Medvik), MEDLINE (OvidSP), Pedro, ProQuest Central, PsycINFO, Scopus, and Web of Science. Grey literature source searches included Clinical Trials, Current Controlled Trials, and Google Scholar. Hand searches were conducted in reference lists of included articles as well as in the journals “Voices: A world forum for music therapy,” “Approaches: An interdisciplinary journal of music therapy,” “Music and Medicine: An interdisciplinary journal” and books “Music vibration and health” and “The art and science of music therapy.” The search was conducted for the period 1980 to March 2021. The search was conducted by an experienced information specialist/librarian (ZS), after preliminary searches had been conducted by JK. Three full electronic search strategies are shown in Appendix A as examples.

### Methodological Quality and Data Extraction

After the search was completed, all citations were collated and uploaded to Zotero 5.0 (Roy Rosenzweig Center for History and New Media, George Mason University, Fairfax, VA, United States) and duplicates were removed. The titles and abstracts were screened by two independent reviewers (EAC and JK) according to the inclusion criteria. The full texts of potentially relevant articles were retrieved and independently screened by the two reviewers. The reference lists of all relevant studies were also screened for potentially relevant studies. Disagreements that arose were discussed at each stage, with input from a third reviewer if unresolved.

Articles included were also assessed by two independent reviewers (JK and LK) for methodological quality and checked by a third reviewer (EAC). A critical appraisal according to the JBI Critical Appraisal Tools (Lockwood et al., 2015; Moola et al., 2020; Tufanaru et al., 2017) was conducted. Although not standard in a scoping review, a critical appraisal was conducted to identify the quality of the included studies, since low quality studies may point towards a gap in current knowledge. In a scoping review, Pham et al. (2014) reported critical appraisal methods ranged from subjective assessments made by reviewers to tools for appraising randomised controlled trials. We therefore opted for tools developed for systematic reviews to ensure a rigorous process. Data were extracted using the data extraction tool developed by the authors (see Appendix B). The extracted data included the article title, author and publication year, country and setting, the design of the study/description of the experiment, including whether there was a sham, blinding, the method of allocation, potential methodological challenges mentioned by the authors, as well as the research sample, type of dementia, potential comorbidities, participant and intervention characteristics (Hz applied, device, amplitude, duration etc.) and whether music was included or a therapeutic relationship was a part of the treatment. The music listened to—when reported, as well as primary and secondary outcome measures and outcomes, were also extracted. Additionally, the data extraction tool described in the study protocol (Campbell et al., 2021) was modified to include the objectives, aims, and or hypotheses reported by the authors. The extracted data are presented in tables consistent with the aims of the scoping review (see Tables 1–8 in Appendix D) and are accompanied by a narrative description.

## Results

**Figure 1:**
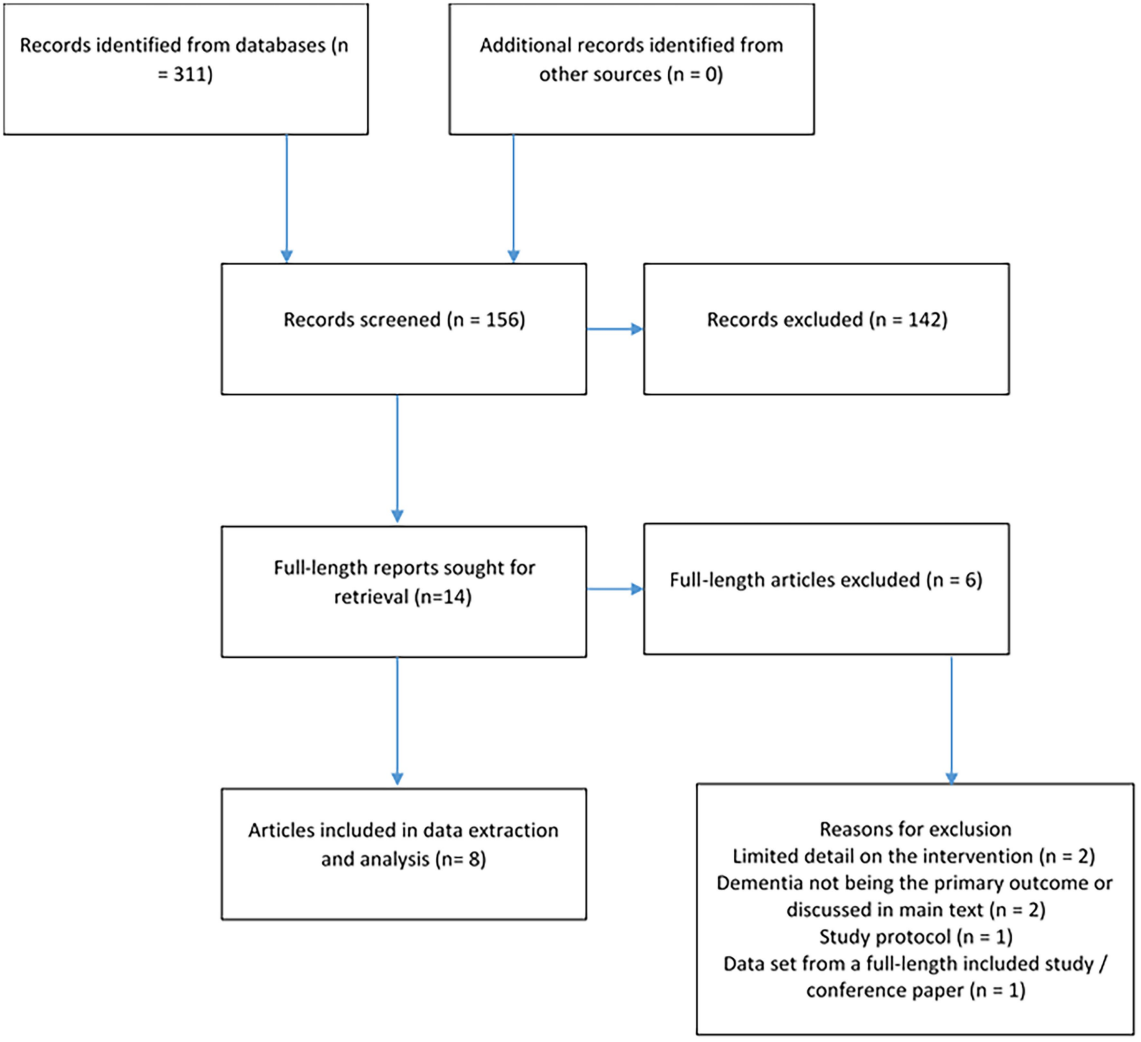
Flow chart of *n* = 8 included studies with *n* = 6 excluded full-text articles for having limited detail on the intervention (*n* = 2), dementia not being the primary outcome or discussed in main text (*n* = 2), for being a study protocol (*n* = 1), and the same data set from a full-length included study (*n* = 1).

The search yielded 311 results and after duplicates had been removed, 156 remained (see Figure 1). Based on titles and abstracts, 142 were excluded for not meeting the eligibility criteria. Fourteen full-length articles were read and assessed according to these criteria; six of these were excluded (see Appendix C in the Supplementary Material for list of excluded studies) for not having enough detail on the low frequency vibration aspect of the intervention (*n* = 2), dementia not being the primary outcome or not being discussed in the article’s main text (*n* = 2), for being a study protocol (*n* = 1), and for the dataset from an included article in the form of a conference paper (*n* = 1). A total of eight studies published between 1993 and 2019 (see study characteristics in Table 1) were included in the analysis and were critically appraised (see section Study Quality Appraisal).

**Table 1:** Study characteristics and main outcomes.

Author and year	Title	Design	Intervention characteristics	Outcomes
Clair and Bernstein, 1993	The preference for vibrotactile vs. auditory stimuli in severely regressed persons with dementia of the Alzheimer’s type compared to those with dementia due to alcohol abuse.	Quasi-experimental study	Vibrotactile (sound vibration) stimulation delivered through Somatron bed Vibration: music generated No therapeutic relationship Further characteristics (e.g., Hz) not specified	No trends in stimulus preference Statistically significant durations in first test pair (study design artefact)
Clements-Cortes et al., 2016	Short-term effects of rhythmic sensory stimulation in Alzheimer’s disease: an exploratory pilot study.	Randomised controlled trial	Comparison of low frequency sound vibration (Next Wave Physioacoustic Chair) and DVD	Positive improvement in vibration group; vibration stimulated participants; DVD had a sedative effect
Clements-Cortes et al., 2017a	Can rhythmic sensory stimulation decrease cognitive decline in Alzheimer’s disease? A clinical case study.	Case report	12 sound vibration sessions with Next Wave Physioacoustic chair (40 Hz, 4 s cycle, 104–109 dBc, 12–45 min sessions, ambient music in five sessions) followed by daily at-home sessions for 3 years (Energise programme mix of vibration and music, 30–60 min/day, focus on 40 Hz)	After 3 years, MMSE score 22/30 (typical decline is 3.3 points per year) 0.5 point SLUMS score effect per treatment Frustration subsided, can still remember children’s names
Clements-Cortes et al., 2017b	The potential of rhythmic sensory stimulation treatments for persons with Alzheimer’s disease.	Qualitative research	Qualitative dataset from Clements-Cortes et al. (2016) (sound vibration vs. DVD crossover study comparing three stages of dementia)	Mild participants more alert in VAT group, boredom/anxiety in DVD group Moderate: increased arousal, alertness in VAT group; DVD: confusion, sleepiness, anxiety Severe: less engagement in both treatments, less coherent verbal communication Greater evidence of verbal communication in VAT group compared to DVD group
Heesterbeek et al., 2019a	Feasibility of three novel forms of passive exercise in a multisensory environment in vulnerable institutionalised older adults with dementia.	Randomised controlled trial	Mechanical vibration delivered through Pactive Motion device 30 Hz, amplitude 1–2 mm, 4 min Comparison of mechanical vibration (WBV) vs. vibration matched to video (TMSim), their combination (WBV + TMSim) and control group	Adherence highest in TMSim + WBV group 52 of 90 participants (able to judge sessions) indicated they were pleasant Some motion sickness reported
Kim and Lee, 2018	The effects of whole body vibration exercise intervention on electroencephalogram activation and cognitive function in women with senile dementia	Quasi-experimental study	Mechanical vibration delivered through VM-10 device, passive and active exercise on the device (e.g., squats, standing) 20 Hz, increased 5 Hz every 2 weeks (max. 35 Hz), 5 times per week, 8 weeks (total 40 sessions)	EEG activation—significant improvement Significant improvement in MMSE-K
Lam et al., 2018	Effects of adding whole-body vibration to routine day activity programme on physical functioning in elderly with mild or moderate dementia: a randomised controlled trial	Randomised controlled trial	Mechanical vibration delivered with Pro 5 Power Plate, 30 Hz, 2 mm amplitude, 2/week, standing on platform, static and dynamic semi-squats	Significant mobility and balance improvements in both groups TUG improved at follow-up but not post-training Balance and Tinetti tests significantly higher post-training QoL-AD reduction post-training and follow-up High attendance rate and low adverse events rate
Mercado and Mercado, 2006	A programme using environmental manipulation, music therapy activities, and the Somatron^©^ vibroacoustic chair to reduce agitation behaviours of nursing home residents with psychiatric disorders.	Case report	Sound vibration delivered using Somatron clinical recliner, EZ Access Model; 2/weekly 30 min sessions including music listening Further characteristics (e.g., Hz) not described	Reduction “as needed” and “give immediately” medication Decrease in some recorded behaviours (e.g., pulling up dress); increase in others (keeping eyes closed during Somatron), increase in verbalisations during sessions Development of the Somatron matrix—inclusion criteria specifier based on presented symptoms

Out of the eight included papers, five studies examined the use of sound vibration and three studies investigated mechanical vibration. The mechanical vibration studies were all more recent publications (2018–2019). Three studies were from Canada, two from the United States, and one each from Hong Kong, Korea, and The Netherlands. The studies were conducted in various contexts; the sound vibration studies were conducted in a Veterans Medical residential centre (Clair and Bernstein, 1993), an in- and out-patient facility (Clements-Cortes et al., 2016, 2017b), a long-term facility (Clements-Cortes et al., 2017a), and a psychiatric nursing facility (Mercado and Mercado, 2006). The mechanical vibration studies were carried out in a nursing home (Heesterbeek et al., 2019a) or day-care centre (Lam et al., 2018) and with community-dwelling participants (Kim and Lee, 2018). Participants ranged from 45 to 103 years old. The study from the Netherlands had the most participants (*N* = 120). All studies included both male and female participants. In most studies the exact dementia diagnosis (in some cases it was not a confirmed diagnosis) was not defined; where mentioned, Alzheimer’s disease was the most common form.

In terms of design, there were three randomised controlled trials (Clements-Cortes et al., 2016; Lam et al., 2018; Heesterbeek et al., 2019a), two quasi-experimental studies (Clair and Bernstein, 1993; Kim and Lee, 2018), two case studies (Mercado and Mercado, 2006; Clements-Cortes et al., 2017a), and one qualitative research study (Clements-Cortes et al., 2017b). One grey literature source (preprint) was located (Heesterbeek et al., 2019b) but was not included in the formal analysis. Conducted in the same setting as Heesterbeek et al. (2019a), results found no significant results of passive exercise on primary outcomes quality of life (QUALIDEM and EQ-5D-5L) nor activities of daily living (Barthel Index). Furthermore, no meaningful effects were found for cognition (Mini mental state examination; MMSE) or balance-related outcomes (Timed Up and Go). Since the manuscript is not published, and in line with the publisher’s requirements, the full methods and results of this source are not described in detail.

### Study Quality Appraisal

Eight articles were critically appraised (see Table 9, Appendix D) by two independent reviewers (JK and LK) and verified by a third independent reviewer (EAC) using the JBI Critical Appraisal Tools (Lockwood et al., 2015; Moola et al., 2020; Tufanaru et al., 2017). Although there is no scoring method applied in the JBI critical appraisal tool, the following approach was used to offer a means of summarising the results: *no* was scored as 1, *unclear* as 2, *yes* as 3. If an item was not relevant to the study, it was not calculated in the total score. The points were added and a percentage calculated according to the possible total. Using this scoring system, the study quality ranged from 58.33% (case report) to 88% (quasi-experimental study).

Three studies investigated mechanical vibration and five addressed sound vibration; the mechanical vibration studies were generally of higher quality than the sound vibration reports. This is especially seen in the description of the treatment/stimulus; in all three mechanical vibration studies, the stimulus is described in a standard manner. This is not the case for the sound vibration studies. The randomised controlled trials were higher quality than the case reports or qualitative study, however essential information such as randomisation methods, allocation concealment, or participant, assessor and interventionist blinding were not reported as well as having issues with measurement reliability. Furthermore, neither follow-up nor intention-to-treat analysis were consistently completed. Issues in the quasi-experimental studies related to lack of clarity of other treatment exposure, reliability of outcome measurements, the statistics, as well as design bias. For example, in the case of Clair and Bernstein (1993), the study investigated participant stimulus choice however participants were incapable of making the decision due to cognitive impairment. The sample sizes in the quantitative studies were also small, which may influence the interpretation power of the results. The case studies were rather low quality; basic demographic information/patient histories were missing as well as a sufficient description of the results and patient assessment, the current and post-treatment clinical condition, adverse events, the treatment procedures, and a takeaway lesson. The qualitative study lacked congruence in methodology with the research question as well as a statement locating the researcher culturally or theoretically and the researcher’s reflection of their potential impact on the research. Overall, participants’ reflections on the treatment are also somewhat lacking however this was reported in the Mercado and Mercado (2006) and Heesterbeek et al. (2019a) studies. Generally, studies lacked participant or outcomes assessor blinding.

### Participant Responses to the Low Frequency Vibration Interventions

In response to review question 1, different types of outcome measures were used to measure participant responses. Neuroimaging was used in one study (electroencephalogram; EEG) (Kim and Lee, 2018). Questionnaires used in the mechanical vibration studies assessed functioning (Timed-up-and-go test; Tinetti test; Berg Balance Scale; Kim and Lee, 2018) as the aims were focused on its use as a form of passive exercise (Heesterbeek et al., 2019a); however cognitive tests were more common in the sound vibration studies. The Saint Louis University Mental Status (SLUMS) exam, a 30-point, 11-question assessment tool for mild cognitive impairment, was used in three studies (Clements-Cortes et al., 2016, 2017a,b); the Mini Mental State Examination (MMSE), a 30-point questionnaire used in clinical and research settings to measure and screen for cognitive impairment/dementia, or its translation, was used in four studies (Clements-Cortes et al., 2017a; Kim and Lee, 2018; Lam et al., 2018; Heesterbeek et al., 2019a). Other types of observations included preference for a particular stimulus (Clair and Bernstein, 1993), researcher observation and the Observed Emotion Rating Scale (Clements-Cortes et al., 2016, 2017b), adherence/attendance rates (Lam et al., 2018; Heesterbeek et al., 2019a) and participants’ subjective experience scores (Heesterbeek et al., 2019a) as well as medication patterns and staff absences (Mercado and Mercado, 2006) and video recordings (Clair and Bernstein, 1993). One study had neither pre–post measurements nor follow-up as the outcomes were only measured during the sessions (Clair and Bernstein, 1993). All questionnaire-based measurements were completed pre- and post-intervention; follow-up was completed in Lam et al. (2018) at 3 months and after 3 years in Clements-Cortes et al. (2017a). Better outcomes were reported in studies where the stimulation was delivered more frequently. For example, in the sound vibration studies, Clements-Cortes et al. (2017a) reported daily use for 30–60 min per day for 3 years and stable cognition throughout this period; MMSE usually scores decline annually by 3.3 points. Of note for the home-based intervention is that the tactile stimulus is combined with auditory stimulus (i.e., music) and as such cannot be separated from the sound vibration effects. In the mechanical vibration studies, sessions were offered five times per week for 8 weeks with statistically significant improvement in EEG activation as well as the Korean translation of MMSE (Kim and Lee, 2018). Unfortunately, a comparison in neuroimaging results between mechanical and sound vibration is not possible since only behavioural/psychological questionnaires were used in the latter.

The 40-Hz sound vibration stimulation improved cognition in mild, moderate, and severe AD participants in Clements-Cortes et al. (2016). The results indicate the increased SLUMS scores for 40Hz diminish with disease severity, however this was statistically insignificant and results were nevertheless significant. Alternatively, in the mechanical vibration studies, the sample size was not large enough to conduct subgroup analyses to compare the impact of WBV on mild and moderate dementia (Lam et al., 2018). BPSD was not assessed by Heesterbeek et al. (2019a), however, even in these severe cases, attendance was still high and participants indicated the sessions were pleasant. EEG activation was significantly improved in mild dementia (Kim and Lee, 2018). These results may indicate that the intervention, although pleasant for those in the later stages of dementia, may be less effective for slowing cognitive decline. However, as mentioned by Clements-Cortes et al. (2016), accurately measuring small changes in cognition is problematic when only questionnaires are used and neuroimaging to supplement these outcomes is necessary. Still, the qualitative outcomes supported the quantitative results in the sound vibration versus DVD (control group) comparison. The qualitative findings showed the control intervention had a sedative effect on participants as well as increasing agitation, boredom, and tiredness. In the sound vibration group, participants had increased awareness of their surroundings, were stimulated to engage in discussions or storytelling and had increased interaction, and were generally more alert. The authors reported that sound vibration appeared to have the largest effect on participants with mild to moderate Alzheimer’s disease. Medication patterns and staff absences were also measured in one study (Mercado and Mercado, 2006); there was a 91% reduction in medication “as needed,” and a 36% reduction in medication required immediately. During the three-month baseline period, there were 482 calls from staff members requesting unplanned absences which reduced to 270 at the conclusion of the intervention, indicating the general atmosphere was also more pleasant for staff.

### Intervention Characteristics Reported in Sound and Vibration Interventions

In answer to review question 2 on the intervention characteristics in the reported studies and how these compare and differ across approaches, the duration of the sessions ranged from 12 (Heesterbeek et al., 2019a) to 45 min (Clements-Cortes et al., 2016; Kim and Lee, 2018); the frequency ranged from daily (Clements-Cortes et al., 2017a) to twice per week (Clements-Cortes et al., 2016). The shortest therapy duration was 6 weeks and the longest was 3 years (self-administered; Clements-Cortes et al., 2017a). A Somatron device was used in two sound vibration studies; in Clair and Bernstein (1993), a Somatron bed was used; in Mercado and Mercado (2006), the EZ Access Clinical Recliner was used. The vibration was dependent on the music source, i.e., the music generates a vibration sensation when played through the device and the specifications of the vibration stimulation therefore cannot be distinguished from the music. The duration of the treatment was based on the duration of the music however this was not specified in these studies. The specific frequencies (Hz) were music dependent and varied according to the music stimulus and as such were not described. Furthermore, amplitude, pulsation or cycle duration were also not described. The music choices which determined the vibrotactile sensation, however, were described. These included classical, country, and an East Indian drone. Mercado and Mercado (2006) offered the stimulus as part of music therapy whereas Clair and Bernstein (1993) did not include a therapeutic relationship and was a purely experimental study. In the remaining three sound vibration studies, the Next Wave Physioacoustic recliner chair was used as well as the VTS1000 Sound Oasis vibroacoustic device for the home-based, self-administered sessions. This device is placed behind one’s back and its dimensions are 13.97 × 45.21 × 78.23 cm. In the Next Wave sessions, 40 Hz was used with an amplitude cycle of 2.3 s (Clements-Cortes et al., 2016, 2017b) or 4 s (Clements-Cortes et al., 2017a), the time in which the stimulus increased from silence to the designated amplitude and returned to silence. The stimulation moved progressively through the six speakers from legs to head and back again (Clements-Cortes et al., 2016, 2017b) or alternating every 2 min between constant, head to legs, and legs to head in cycles throughout the stimulus (Clements-Cortes et al., 2017a). The home-based stimulation delivered by the VTS1000 (Clements-Cortes et al., 2017a) included a 60-min programme called “Energise” which consists of ~20 min of 40-Hz stimulation with 4-s cycle amplitude which is mixed with/generated from “relaxing ambient music.” The exact specifications of the stimulus and what other frequencies were in the programme are not reported.

The characteristics of the mechanical vibration studies were more systematically described although the stimulus was delivered using three different devices in each of the three studies. Heesterbeek et al. (2019a) used the Pactive Motion whole body vibration device; Kim and Lee (2018) used the VM-10; and Lam et al. (2018) used the Pro 5 Power Plate device. All three devices deliver whole body stimulation, either with the participant standing or sitting in a wheelchair on a vibrating platform. The mechanical vibration interventions are generally shorter than the sound vibration, however the nature of the intervention is different. Rather than being a constant stimulus, and given the exercise-oriented nature of the intervention, there are bouts of vibration followed by rest periods for a period lasting 12 (Heesterbeek et al., 2019a) to 40 min (Kim and Lee, 2018). Kim and Lee (2018) increased the Hz over time so that it began with 20 Hz and progressed to 35 Hz by the end of the 8-week training period. Lam et al. (2018) increased the duration of the vibration, beginning with 30 s bouts and ending with 45 s bouts at the end of the 9-week programme however the frequency remained at 30 Hz throughout. Heesterbeek et al. (2019a) also used only 30 Hz throughout. Participants simultaneously engaged in either static or dynamic exercises (Kim and Lee, 2018; Lam et al., 2018) or passively received the vibration whilst in their wheelchairs (Heesterbeek et al., 2019a). The amplitude in the mechanical vibration studies was measured in mm rather than in dB as in the sound vibration studies (Heesterbeek et al., 2019a, 1–2 mm; Lam et al., 2018, 2 mm; not mentioned in Kim and Lee, 2018). There was no therapeutic relationship involved in the mechanical vibration studies; the nature of these interventions was a physical focus rather than the emotional/cognitive focus potentially more distinctive of the sound vibration studies. It was clear in the mechanical vibration studies, that the treatment parameters (e.g., Hz) developed over time which was not the case in the sound vibration studies. No rationale is provided for the progressive increase in Hz during the training period.

### Specifics of the Research Experiments

In answer to review question 3 on the specifics of the research experiments in studies on tactile low frequency vibration and dementia, the included studies comprised of three randomised controlled trials (RCTs), two quasi-experimental studies, two case reports, and one qualitative study. The qualitative study was not situated within a specific paradigm.

The studies reported various objectives and rationales. Both sound and mechanical vibration studies aimed to increase cognitive function and alertness however the rationale was based on 40 Hz increasing gamma coherence—which is said to support intracerebral communication—in the sound vibration studies (Clements-Cortes et al., 2016, 2017a,b) and to increase brain metabolism, cerebral blood flow and neurotransmitter secretion (Kim and Lee, 2018). In the mechanical studies, the rationale was to reduce pathology severity and disease progression through passive/semi-passive exercise (Heesterbeek et al., 2019a) in the mechanical studies. In the one study (Mercado and Mercado, 2006) in which a therapeutic relationship was part of the intervention, the rationale was that environmental factors have been shown to increase agitation in this population and active participation in individualised music has been successful in meeting a variety of needs, facilitating behaviour management and increasing social and cognitive skills and self-esteem whilst interventions including vibrotactile stimulation had increased participation from persons with Alzheimer’s disease. Three studies were feasibility studies (two feasibility studies and one pilot/proof of concept study) and the qualitative study aimed to further the understanding of how low frequency sound vibration can improve cognition in this population.

Issues which arose due to design and study population included severity of disease (Heesterbeek et al., 2019a) leading to inclusion bias, since more frail participants were excluded as a result of protective legal representatives withholding permission and the potential for overstimulation (possibly due to inability to express oneself coherently). Dizziness from the stimulation, potentially less potent effect from the stimulation due to less than ideal frequency (twice rather than three or more times per week; Clements-Cortes et al., 2016, 2017b), refusal to attend the sessions (Mercado and Mercado, 2006), non-standardised interview questions and potential subjectivity of the therapist or their observations and assessments (Clements-Cortes et al., 2017a), and difficulty in measuring incremental changes in cognition (Clements-Cortes et al., 2016) were all reported.

Five studies used some form of a control group(s). In the RCTs, Clements-Cortes et al. (2016) compared low frequency sound vibration with a DVD in a randomised crossover pilot study. Participants therefore could not be blinded but were randomised into their primary intervention group. Heesterbeek et al. (2019a) had three intervention arms and a control group. The interventions compared multisensory stimuli (whole body vibration, therapeutic motion stimulation, or their combination) whilst the control group received standard care. Participants in Kim and Lee (2018) also received their usual care routine. Although the study was described as single blind (Heesterbeek et al., 2019a), it was not clear who was blinded. In many cases, blinding was potentially impossible because participants can feel the stimulation. However, the potential of using a sham device to simulate the vibration—possibly by hearing the auditory element of the low frequency vibration but not feeling it—was not discussed. As for the quasi-experimental studies, Clair and Bernstein (1993) did not have a control group however one condition of the experiment was silence, meaning no stimulation. The procedures for the control group were not mentioned in Kim and Lee (2018) although this is presumed to have been continuing with standard procedures of daily life since the study was carried out with community-dwelling participants. Participants in the other studies were not community-dwelling, so a direct comparison of their standard procedures is not possible. Standard care across the remaining articles is presumed to include medication and other therapies; an exact description of the usual care procedures was not available.

### Novel Characteristics of the Studies

Although not a direct objective of the study, the Somatron Matrix developed by Mercado and Mercado (2006) takes a first step towards needs-based assessment and the potential clinical applications of low frequency interventions for dementia. It is an example of novel aspects of the reported interventions. The matrix addressed various criteria such as to what extent the person exhibited verbal communication, their agitation level, whether they could follow directions, had an observed need for relaxation, and the proposed potentially useful intervention (i.e., music therapy or vibration or their combination), frequency, some characteristics of the Somatron intervention, e.g., therapist modelling relaxing behaviours such as closing eyes, observations to be made (e.g., physiological changes, movements), and the music considerations. This was necessary due to the wide range in functioning and behaviours exhibited by the nursing home residents and the need for more specific inclusion criteria. Its purpose was to streamline if and in what capacity the sound vibration should be applied. For example, if the client displayed limited or no response to stimuli, Somatron sessions were delivered three times a week, with the therapist having close verbal, olfactory, and tactile stimulation during the intervention, making observations on physiological changes such as heart rate and blood pressure, with input from caregivers or family for the music choices. Each of these aspects was scalable depending on the needs of the client. This matrix offers the possibility to categorise for whom and under what conditions the Somatron may be beneficial and possibly tailoring the, e.g., frequency of the sessions as the client’s stimuli response capabilities develop over time. Although the authors reported there were common characteristics determining which persons benefited more from each of the interventions, these are not explicitly disclosed. However, diagnosis apparently did not factor in this assessment. As mentioned by Heesterbeek et al. (2019a), a low frequency intervention is tailorable to the client’s needs in terms of, e.g., duration or intensity, which offers flexibility in its application.

## Discussion

This scoping review has explored the use of sound and mechanical low frequency vibration interventions in dementia management. Of the eight included studies, five were sound vibration-based and the remaining three were mechanical vibration studies. There were three RCTs, two quasi-experimental studies, two case studies, and one qualitative research study. Study design, outcomes and outcome measures, objectives, and participant and intervention characteristics varied across the studies. The extracted data show the studies in this area are also heterogeneous in terms of quality and standards of reporting. The findings in this scoping review support those of Monteiro et al. (2021) that there is no evidence of comparison between treatment specifications which leads to suboptimal and distinctive treatment/experimental protocols. Bunn et al. (2014) reported that there is a high prevalence of comorbid conditions in persons with dementia, however this was not discussed in the reviewed studies. Only one of the mechanical vibration studies reported the comorbid diagnoses at all and the demographic data or anamneses were otherwise sparse. This is valuable information (Mosabbir et al., 2020) which contributes to the understanding of how an intervention may work for a particular population and more importantly offer insights into why an intervention may be contraindicated or not beneficial. Many studies did not include basic demographic information including dementia diagnosis.

### Methodological Issues

Methodological issues in these studies include the control group and blinding. Since it may be presumed that any intervention is more beneficial than no intervention, comparing sound or mechanical vibration to a sham vibration stimulus would ensure a control comparison as well as enable blinding. In one low frequency sound vibration study investigating the effects on recovery from exercise-induced muscle damage, a sham intervention was used in which participants sat in the Next Wave Physioacoustic chair and believed they received the stimulation but the device was turned off (Tiidus et al., 2008). However, it was also reported that participants in this group also received a few training sessions sitting on the device prior to the data collection. In a recent study, Mosabbir et al. (2020) had a sham control group in which the participants could hear the acoustically simulated 40 Hz hum, but it was not delivered through the device. They were furthermore told they would not necessarily feel the vibration stimulus, thus ensuring blinding remained intact. In nursing home residents with low physical functioning partaking in an exercise programme, Sievänen et al. (2014) compared exercising on the whole-body mechanical vibration platform with and without the added vibration stimulus, referring to the latter condition as the sham. It is not clear whether participants were blinded to this or not. Comparing several groups such as was reported by Heesterbeek et al. (2019a) solves the direct intervention versus standard care control group issue however having multiple intervention groups also increases the expense and still does not allow for blinding. Potentially problematic in the Clements-Cortes et al. crossover study (2016) is that the duration of effect as measured by follow-up is unknown and the potential carry-over effect is therefore to be considered, although, in this case, a direct individual comparison between the interventions is possible.

The low number of relevant studies included in the analysis shows that despite most of the articles being published in the last 5 years, there are still gaps in the literature in terms of study designs comprehensively addressing participants’ responses to the intervention. However, as reported in Heesterbeek et al. (2019a), participants were not always capable of giving their opinions and in the case of Clair and Bernstein (1993), participants were generally incapable of making decisions thereby impacting how the effectiveness of the intervention is interpreted. These participants generally had more severe dementia owing to their need for institutional care; comparably clearer outcomes are seen in, e.g., Kim and Lee (2018) in which the participants were community-dwelling and had only mild dementia. Therefore, the severity of the disease precludes gleaning more insight into participants’ subjective responses to low frequency vibration interventions. Heesterbeek et al. (2019a) also reported that frail older adults are generally excluded from study participation due to reduced cognitive function, poor mobility and comorbidities, as well as legal representatives preventing participation. This also leads to difficulties in investigating appropriate interventions for the more vulnerable persons in this target group.

### Quality of Intervention Reporting

Although the reporting in the mechanical vibration studies was partly more standardised compared to the sound vibration studies, important details—such as whether the WBV platform was vertical or horizontal—were missing. The training and qualifications of persons delivering the interventions should be standardised, intervention characteristics, device descriptions (e.g., WBV—vertical or horizontal vibrating platform), who is delivering the treatment and with which qualifications this treatment is being offered (van Heuvelen et al., 2021). According to Wigram and Dileo (1997), as vibroacoustic therapy is a form of treatment, it should be delivered by clinicians who have appropriate knowledge and experience or be offered under a supervision with relevant qualifications. Furthermore, the authors highlight that appropriate application also requires enough theoretical knowledge of its scientific basis as well as a reliable procedure for its application. With this in mind, although music therapists delivered the sound vibration interventions, the type of training received as well as the exact procedures of the intervention were not specified. This should be standardised, i.e., guidelines should be developed to support this, especially when investigating the efficacy or effectiveness of an intervention in a field with lacking research such as dementia. Similarly, in the mechanical vibration studies, it was not reported who was delivering the intervention and if/what training they had. Vibroacoustic therapy—a low frequency sound vibration intervention—is described as a receptive music therapy intervention due to the combination music experiences within a therapist-client relationship (Ala-Ruona and Punkanen, 2017; Campbell, 2019). Despite the intervention being delivered by a music therapist in four of the five sound vibration studies (Mercado and Mercado, 2006; Clements-Cortes et al., 2016, 2017a,b), the interventions themselves were not sufficiently described to enable comparison with other music therapy studies.

### Intervention Characteristics Requiring Future Research

It appears that more frequent sessions led to better outcomes. In studies with sessions five times per week, outcomes were more distinctive compared to those only offered twice per week. Indeed, the home-based intervention described in Clements-Cortes et al. (2017a) suggests that this may prevent disease progression when applied daily. This may indicate that in order to ensure sustained results, more frequent vibration stimulation is required but that self-administration is also beneficial. Long-term mechanical vibration interventions (5 days—5 weeks) using lower frequencies (between 15 and 30 Hz) were reported in one systematic review (Dincher et al., 2019) to lead to greater improvement in Parkinson’s disease symptoms. In another systematic review (Lam et al., 2012), frequencies between 12.6 and 26 Hz produced significant results whilst those ranging from 35 to 40 Hz did not. The authors explained that the difference in treatment effect is likely due to the protocol itself, because of the variation in transmissibility dependent on the specific combinations of frequencies and amplitudes. Indeed, the three studies included in this scoping review did not include stable frequencies throughout their protocols. In comparison to the sound vibration interventions, the mechanical vibration sessions are generally short (12 min) which may enable one to attend the sessions more often. On the other hand, the length of the sessions may not positively impact the psychosocial aspects of dementia, given that there is a lack of a therapeutic relationship in the reported mechanical vibration interventions. The lack of therapeutic relationship is perhaps based on the differing rationales between the two types of interventions. Mechanical vibration studies leaned towards physical /physiological outcomes whereas the sound vibration studies focused on the psychological/cognitive aspects of dementia. Although the rationale for the Clements-Cortes et al. studies (Clements-Cortes et al., 2016, 2017a,b) was based on cerebral coherence at the 40-Hz gamma level, it was not possible to address this issue in the design used. Kim and Lee (2018), for example, showed that participants’ EEG activation increased after the mechanical vibration intervention and used the appropriate method to explore this hypothesis. Similar results are seen in Jeong et al. (2021) with healthy older adults in which increased EEG activation was reported in both WBV in an upright stance compared to WBV with a squat stance.

Authors’ recommendations for future research included larger sample sizes, using neuroimaging to measure cerebral coherence from sound vibration, increasing intervention frequency, with authors also acknowledging the limitations of the statistical conclusions and the potential subjectivity of the therapist and their observations due to non-standardised interview questions. Bartel and Mosabbir (2021), in a narrative review of mechanical and sound vibration and its underlying mechanisms, also suggest the addition of the following to increase clarity of reporting: describing the applied frequency rather than referring to it as “high” or “low” frequency; reporting the surface area/region of the body directly stimulated; and a standard base of reporting the frequency (Hz), acceleration (in *g*), duration of stimulus (time applied and over number of days); and the area of body that is stimulated. As discussed by Wuestefeld et al. (2020), correct terminology—especially due to the interdisciplinarity of the field—is needed to ensure high-quality studies and reporting so that findings can be compared in systematic reviews. Inconsistent terminology also leads to lack of knowledge on the potential effects of the stimulus and is such is also essential for improving the clinical outcomes.

A potential solution to the feasibility of delivering more frequent treatments was found in Clements-Cortes et al. (2017a) in which the participant received 12 sessions with the therapist followed by daily home-use of a commercial device. It may be that the initial sessions offer a foundation and that the continued home-based treatments, which can be delivered much more frequently, sustain, support, or continue the initial outcomes. This was found, for example, in sound vibration applications in chronic pain management (Campbell et al., 2019). Self-applied, home-based VAT for Ehlers-Danlos syndrome in an N-of-1 trial also showed modest improvements after daily treatments for 4 weeks and worsening in washout post-treatment (Picard et al., 2018).

### Theoretical Framework of the Interventions

Although vibroacoustic therapy may be considered a receptive music therapy method (Hooper, 2001; Grocke and Wigram, 2006), only two of five studies (Clair and Bernstein, 1993; Mercado and Mercado, 2006) seemed to apply the technique within a session described as a music therapy intervention. However, the intervention was offered by a trained music therapist in the remaining three studies, which speaks to clinical and ethical applications of the intervention. The theoretical framework of the mechanical vibration studies lay in exercise rehabilitation and did not employ auditory music or a therapeutic relationship; the approach to this intervention is therefore different than the psychologically-oriented sound vibration studies.

Yet, since there is at a cellular level probably no difference between oscillation applied through a sine wave directed to the body or by an oscillating surface with contact to the body, the underlying mechanisms are potentially the same (Bartel and Mosabbir, 2021). Bartel and Mosabbir discussed the impact of vibration on cognitive impairment from two angles. Firstly, since many diseases are linked to oxidative stress (including Alzheimer’s and Parkinson’s diseases) and pulsed stimulation can significantly increase antioxidant expression, vibration may thus indirectly improve cognitive functioning. Secondly, although the causes or mechanisms of neurodegenerative diseases are not well understood, brain oscillation/dysrhythmia is being explored. Dysregulated 40 Hz, i.e., gamma activity, is reported in Alzheimer’s patients, which was reported to improve after applied 40-Hz auditory, vibrotactile/visual rhythmic sensory stimulation. Although not confirmed with brain imaging, the authors propose the results are inferable from the method and results.

### Strengths of the Scoping Review

This review offers insights into the overlaps and differences in reporting in the research and clinical applications of tactile low frequency vibration. It is the first report to the author’s knowledge comparing sound and mechanical vibration in dementia care. Research has been increasing in both areas in recent years and as such transferrable and comparable reporting standards are necessary to improve clarity and allow for replication. Understanding what research outcomes and its clinical applications are reported allows for improved quality and ultimately leads towards improved standards of care. Non-pharmacological interventions are necessary to manage the symptoms of dementia and tactile low frequency vibration may be potentially beneficial. The strength of the current review therefore lies in bringing together similar interventions from disparate applications and presenting the strengths and weaknesses to improve future work and potentially enable exchange between those researching and practicing in the respective areas.

## Conclusion

In this scoping review, we found studies reporting that low frequency sinusoidal sound and mechanical vibration interventions may increase EEG activation and cognitive function as well as alertness and arousal and that these interventions may decrease deviant motor behaviours and the impact on those supporting people with dementia. This review also showed there was no crossover in reporting and intervention characteristics between sound and mechanical vibration, despite similar characteristics of the stimulus itself. In accordance with the research questions guiding this review, participants’ subjective responses to these interventions require further investigation in a manner which can meaningfully represent those of people with dementia. The intervention can be tailored and adapted according to the client’s specific needs and this should be adhered to in delivering this intervention. Results also seemed to indicate that more frequent applications of the stimulus—even in a self-delivered, home-based setting—have greater impact on symptoms, however, further study is required to delineate the specific parameters of the interventions which may lead to effective dementia symptom management. A more detailed understanding of how vibration impacts dementia symptoms is warranted, with a discussion on the differences or similarities between various vibration-based methods and their characteristics. Finally, following structured guidelines in reporting interventions such as those set forth by van Heuvelen et al. (2021) and Wuestefeld et al. (2020) is needed to enable study replication and comparison across methods.

### Limitations

This scoping review had several limitations. Firstly, the low transparency in the studies included in the review along with the limited number of papers and the methodological challenges of some means that the scope of possible outcomes may not been sufficiently investigated. Due to the relatively low quality of the included studies, caution is needed in interpreting the outcomes reported. The sample sizes in the studies were relatively small (only one study had more than 100 participants), meaning the statistical significance of the results should be interpreted with caution. The descriptions of the interventions in the sound vibration studies were also rather sparse and allow neither for replication nor for gleaning deeper understanding of the potential effectiveness of the stimulus. Secondly, papers in other languages in which the title or abstract were not listed in English were not found in our search. It would have been beneficial to be able to differentiate between the effects of each intervention on differing disease severity however only two studies discussed severe dementia. This is problematic given residents of care homes are more likely to have an increased need for care and therefore have more severe dementia. As seen in this scoping review and reported by Moreno-Morales et al. (2020), studies mostly focus on mild or moderate dementia. Finally, although it may be atypical to include case reports in reviews, we wished to be as inclusive as possible to accurately map/scope the field. In this instance, case reports are part of the small pool of evidence on the use of low frequency sound vibration and may be useful in informing the usefulness of conducting RCTs. However, the evidence reported in such case studies is to be interpreted with caution.

### Future Research

As is seen in this scoping review, and supported by the findings of Bartel and Mosabbir (2021), much more research is needed. Furthermore, although the fields of sound and mechanical vibration are separate, there is overlap in, for example, the frequencies used. Each may benefit from each other in terms of intervention planning, conducting, and reporting as well as standardising experimental research. At this time, we do not recommend conducting a systematic review with meta-analysis on the effectiveness of these interventions due to the lack of research in this area and the lack of standardised reporting. We recommend further investigation of the effects of low frequency vibration for the behavioural and psychological symptoms of dementia in the form of RCTs with comparable, standardised methods to enable future systematic reviews with neuroimaging techniques to confirm hypothesised mechanisms, as well as having more focus on participant responses to the treatment. Furthermore, a standardisation of intervention procedures is necessary for ensuring study fidelity and enabling outcomes comparison across studies. Finally, studies should include participants with varying disease severity and large enough sample sizes to enable comparison and sufficient statistical power to draw more concrete conclusions.

## Author Contributions

EAC and JK developed the concept and completed the final draft. EAC drafted the initial manuscript with revisions from JK and LK input from TW. ZS conducted the database searches. JK and LK conducted the initial critical appraisal, with additions from EAC. All authors contributed to the article and approved the submitted version.

## Funding

This research was funded by Deutsche Fernsehlotterie (Deutsches Hilfswerk; application number A2019/229). The Article Processing Charges were furthermore supported by the Concept Evidence-Based Practice in Special Education and Arts Therapies, VaV_PdF_2022_001 and support of mobility at UP II., project number: CZ.02.2.69/0.0/0.0/18_053/0016919.

## Conflict of Interest

The authors declare that the research was conducted in the absence of any commercial or financial relationships that could be construed as a potential conflict of interest.

## Publisher’s Note

All claims expressed in this article are solely those of the authors and do not necessarily represent those of their affiliated organizations, or those of the publisher, the editors and the reviewers. Any product that may be evaluated in this article, or claim that may be made by its manufacturer, is not guaranteed or endorsed by the publisher.
