# Corrigendum: Tactile low frequency vibration in dementia management: A scoping review

**DOI:** 10.3389/fpsyg.2022.1003963

**Published:** 2022-09-16

**Authors:** Elsa A. Campbell, Jirí Kantor, Lucia Kantorová, Zuzana Svobodová, Thomas Wosch

**Affiliations:** ^1^Caritas Association Ettlingen, Ettlingen, Germany; ^2^VIBRAC Skille-Lehikoinen Centre for Vibroacoustic Therapy and Research, Eino Roiha Foundation, Jyväskylä, Finland; ^3^Center of Evidence-Based Education and Arts Therapies: A JBI Affiliated Group, Institute of Special Education Sciences, Faculty of Education, Palacky University, Olomouc, Czechia; ^4^Faculty of Medicine, Czech National Centre for Evidence-Based Healthcare and Knowledge Translation (Cochrane Czech Republic, Czech EBHC: JBI Centre of Excellence, Masaryk University GRADE Centre), Institute of Biostatistics and Analyses, Masaryk University, Brno, Czechia; ^5^Faculty of Health Sciences, Palacky University, Olomouc, Czechia; ^6^Institute for Applied Social Sciences, University of Applied Social Sciences, Würzburg-Schweinfurt, Germany

**Keywords:** low frequency vibration, dementia, vibroacoustic, whole body vibration, scoping review

In the published article, there was an error. The wording was misleading to the results of the cited article, Clements-Cortes et al. (2016). A correction has been made to Participant Responses to the Low Frequency Vibration Interventions section, paragraph two. The corrected paragraph is below.

The 40-Hz sound vibration stimulation improved cognition in mild, moderate, and severe AD participants in Clements-Cortes et al. (2016). The results indicate the increased SLUMS scores for 40 Hz diminish with disease severity, however this was statistically insignificant and results were nevertheless significant. Alternatively, in the mechanical vibration studies, the sample size was not large enough to conduct subgroup analyses to compare the impact of WBV on mild and moderate dementia (Lam et al., 2018). BPSD was not assessed by Heesterbeek et al. (2019a), however, even in these severe cases, attendance was still high and participants indicated the sessions were pleasant. EEG activation was significantly improved in mild dementia (Kim and Lee, 2018). These results may indicate that the intervention, although pleasant for those in the later stages of dementia, may be less effective for slowing cognitive decline. However, as mentioned by Clements-Cortes et al. (2016), accurately measuring small changes in cognition is problematic when only questionnaires are used and neuroimaging to supplement these outcomes is necessary. Still, the qualitative outcomes supported the quantitative results in the sound vibration versus DVD (control group) comparison. The qualitative findings showed the control intervention had a sedative effect on participants as well as increasing agitation, boredom, and tiredness. In the sound vibration group, participants had increased awareness of their surroundings, were stimulated to engage in discussions or storytelling and had increased interaction, and were generally more alert. The authors reported that sound vibration appeared to have the largest effect on participants with mild to moderate Alzheimer's disease. Medication patterns and staff absences were also measured in one study (Mercado and Mercado, 2006); there was a 91% reduction in medication “as needed,” and a 36% reduction in medication required immediately. During the three-month baseline period, there were 482 calls from staff members requesting unplanned absences which reduced to 270 at the conclusion of the intervention, indicating the general atmosphere was also more pleasant for staff.

The authors apologize for this error and state that this does not change the scientific conclusions of the article in any way. The original article has been updated.

## Publisher's note

All claims expressed in this article are solely those of the authors and do not necessarily represent those of their affiliated organizations, or those of the publisher, the editors and the reviewers. Any product that may be evaluated in this article, or claim that may be made by its manufacturer, is not guaranteed or endorsed by the publisher.
