# Supplementary material for: Tactile Low Frequency Vibration in Dementia Management: A Scoping Review
Source: Front Psychol. 2022 Jun 17;13:854794. doi: 10.3389/fpsyg.2022.854794 (PMC9252598; doi:10.3389/fpsyg.2022.854794)
Supplement: Supplementary file 1 [file Data_Sheet_1.pdf]

Appendix A

Examples of the search formula

Table A1. Database: CINAHL Plus with Full Text.

#	search	results		
1	(MH "Dementia+") (Major Headings with Subheadings)	75859		
2	(MM "Alzheimer's Disease") (Major Headings with Subhead	25010		
3	(MM "Lewy Body Disease") (Major Headings with Subheadir	897		
4	(MH "Huntington's Disease") (Major Heading)	1513		
5	Dementia Ti/Ab	54274		
6	Alzheimer* Ti/Ab	35855		
7	Lewy Ti/Ab	2326		
8	Klüver–Bucy syndrome* Ti/Ab	7		
9	Kluver Ti/Ab	37		
10	Huntington Ti/Ab	1766		
10	1-10 OR	98760		
11	vibroacoustic therap* Ti/Ab	9		
12	vibroacoustic music Ti/Ab	2		
13	vibroacoustic sound* Ti/Ab	2		
14	physioacoustic Ti/Ab	1		
15	physio?acoustic sound* Ti/Ab	0		
16	somatron* Ti/Ab	1		
17	low frequency sound stimulation* Ti/Ab	2		
18	vibrotactile stimulation* Ti/Ab	49		
19	rhythmic sensory stimulation* Ti/Ab	6		
20	whole body vibration Ti/Ab	1062		
21	vibration therap* Ti/Ab	149		
22	11-21 OR	1215		
23	10 AND 22	**7**		

Table A2. Database: EMBASE.

#	search	results		
1	'dementia'/exp	376374		
2	'alzheimer disease'/exp	209496		
3	kluver bucy syndrome'/exp	270		
4	´lewy body´/exp	7290		
5	dementia Ti/Ab	164841		
6	´huntington chorea´/exp	27882		
7	alzheimer* Ti/Ab	209996		
8	kluver Ti/Ab	681		
9	huntington Ti/Ab	5376		
10	lewy Ti/Ab	15348		
11	1-10 OR	437432		
12	vibroacoustic therap* Ti/Ab	15		
13	vibroacoustic music Ti/Ab	3		
14	vibroacoustic sound* Ti/Ab	5		
15	physioacoustic Ti/Ab	2		
16	physioacoustic sound* Ti/Ab	0		
17	somatron* Ti/Ab	1		
18	low frequency sound stimulation* Ti/Ab	5		
19	vibrotactile stimulation* Ti/Ab	402		
20	rhythmic sensory stimulation* Ti/Ab	24		
21	vibration therap* Ti/Ab	269		
22	whole body vibration Ti/Ab	2581		
23	12-22 OR	3124		
24	11 AND 23	**13**		

Table A3. Database: PubMed.

#	search	results		
1	Dementia[Mesh]	172075		
2	Alzheimer Disease[Mesh]	97538		
3	Lewy Body Disease[Mesh]	3444		
4	Kluver-Bucy Syndrome[Mesh]	106		
5	Huntington Disease[Mesh]	12374		
6	dementia Ti/Ab	116638		
7	Alzheimer* Ti/Ab	156653		
8	Lewy Ti/Ab	10179		
9	Kluver Ti/Ab	545		
10	Huntington Ti/Ab	18574		
11	1-10 OR	276952		
12	vibroacoustic therap* Ti/Ab	9		
13	vibroacoustic music Ti/Ab	19		
14	vibroacoustic sound* Ti/Ab	5		
15	physioacoustic Ti/Ab	4		
16	physio?acoustic sound* Ti/Ab	1		
17	physioacoustic sound* Ti/Ab	2		
18	somatron* Ti/Ab	1		
19	low frequency sound stimulation* Ti/Ab	6		
20	vibrotactile stimulation* Ti/Ab	363		
21	rhythmic sensory stimulation* Ti/Ab	25		
22	vibration therap* Ti/Ab	216		
23	whole body vibration* Ti/Ab	2145		
24	12-23 OR	2690		
25	11 AND 24	**16**		

Appendix B

Data extraction tool developed by the authors

**Author, year**<br>**Country, setting, language**
Study design / research experiment description<br>(sham, blinding, method of allocation, methodological challenges mentioned by researchers, etc.)<br>Objectives / hypothesis / goals<br>Research sample, type of dementia (diagnosis),<br>co-morbid/ secondary diagnosis(es)<br>Participant characteristics<br>(age, gender, education, profession)<br>Tactile low frequency intervention characteristics<br>(type of vibration [sound, mechanical], frequency (Hz), device, pulsation / cycle duration, amplitude (dB), duration (time), absence/presence of music listening, absence/presence of therapeutic relationship)<br>Music choice<br>Participant / client or therapist chosen music listening<br>Outcome measures<br>Primary outcome measures<br>Secondary outcomes measures<br>Outcomes<br>Primary outcomes<br>Secondary outcomes

Appendix C

List of excluded studies after full-length screening

Clements-Cortes, A,. Ahonen, H., Freedman, M., and Bartel L. (2016) Rhythmic sensory stimulation and Alzheimer's disease. Nord. J. Music Ther. 25,17-17.*Reason for exclusion: Conference paper*

Chung J, Lai C. Snoezelen for dementia (2002). Cochrane Database of Systematic Reviews [Internet]. 4). Available from: http://dx.doi.org/10.1002/14651858.CD003152

Reason for exclusion: Not enough detail on low frequency vibration

Koike, Y., Hoshitani, M., Tabata, Y., Seki, K., Nishimura, R., and Kano, Y. (2012) Effects of Vibroacoustic Therapy on Elderly Nursing Home Residents with Depression.J. Phys. Ther.24,291–294.

Reason for exclusion: Dementia is not the primary outcome / is not discussed in the main text

Park, Y.J., Park, S.W., and Lee, H.S. (2018) Comparison of the Effectiveness of Whole Body Vibration in Stroke Patients: A Meta-Analysis. Biomed Res Int. 2018, https://doi.org/10.1155/2018/5083634.

Reason for exclusion: Dementia is not the primary outcome / is not discussed in the main text

van der Steen, J., Smaling, H., van der Wouden, J., Bruinsma, M., Scholten, R., and Vink, A. (2018) Music-based therapeutic interventions for people with dementia. Cochrane Database of Systematic Reviews [Internet]. 7,. https://doi.org/10.1002/14651858.CD003477.pub4

Reason for exclusion: Not enough detail on low frequency vibration

van Os, A.J., Aziz, L., Schalkwijk, D., Schols, J.M. and de Bie, R.A. (2012). Effectiveness of Physio Acoustic Sound (PAS) therapy in demented nursing home residents with nocturnal restlessness: study protocol for a randomized controlled trial. *Trials*, *13*, 1-7. https://doi.org/10.1186/1745-6215-13-34

Reason for exclusion: Study protocol

Appendix D

Extracted data from included studies

Table 1.

Clair & Bernstein (1993) The preference for vibrotactile versus auditory stimuli in severely regressed persons with dementia of the Alzheimer's type compared to those with dementia due to alcohol abuse

Country, Setting, Language
USA, Veterans Affairs Medical Center, English

Study design / research experiment description *(sham, blinding, method of allocation, methodological challenges mentioned by researchers, etc.)*

- Quasi-experimental study (2.c)
- Sample (9 participants) chosen from pool of 28 patients
- Permission to videotape given from guardians as selection criterion for participation
- Study without control group but a control stimulus (silence)
- Each subject participated in individual testing sessions for 15 minutes three times per week for six weeks for a total of 18 testing sessions. The initial two sessions were practice sessions and were not included in data analysis

Objectives / hypothesis / goals

- Preference of severely regressed persons with Alzheimer's / Dementia for vibrotactile stimuli; addition of vibrotactile stimuli to passive music participation (background music, music offered by nursing staff) could increase participation and promote persons' responses

Research sample, type of dementia (diagnosis), comorbid/ secondary diagnosis(es)

- N = 9 male Veterans in a closed ward requiring total care and continuous supervision
- Alzheimer's disease (n=6) or
- dementia due to alcohol abuse (n=3)
- No dropout mentioned in the study

Participant characteristics
(age, gender, education, profession)

- Male
- 68-75 years old
- Army Veterans
- No further information about the patients' condition before the interventions was provided

Tactile low frequency intervention characteristics
(type of vibration [sound, mechanical], frequency (Hz), device, pulsation / cycle duration, amplitude (dB), duration (time), absence/presence of music listening, absence/presence of therapeutic relationship)

- Sound vibration delivered through the Somatron bed (vibrotactile stimulation through speakers in the mattress)
- Vibrotactile stimulation music-dependent
- Mercury switch attached to arm to affect change in stimuli (vibrotactile stimulation, auditory stimuli, or silence)
- No therapeutic relationship

Music choice

- Tape recording of Patsy Cline's greatest hits - chosen for quieting, sedative qualities and subjects' preference for country music (reported by family members)

Or

- an East Indian drone instrument (tamboura) - chosen for vibrotactile qualities and lack of other musical elements

Both offered as auditory and vibrotactile stimulus (played through Somatron)

Or

- silence.

Each auditory stimulus was combined in all possible pairs, pairs of stimuli chosen for each session were randomised

16 testing sessions in total

Participant/client or therapist chosen music listening

Choice of Patsy Cline, Tamboura, or silence, offered in stimulus pairs designed by the researcher

Outcome measures

Primary outcome measures

- Duration of chosen stimulus and the number of times this was chosen (preference for vibrotactile sound stimulation, non-vibrotactile auditory sound, or silence)

Secondary outcome measures

- Differences in preference between participants with Alzheimer' and dementia from alcohol abuse
- Video recordings were made of each session, but there is no information on the number of raters or intra- / interrater reliability
- No before / after measurements (participants were measured only during the intervention) and no follow up observation was conducted

Outcomes

Primary outcome

- Only statistically significantly longer durations in the first pair (possibly artefact of study design)
- No trends in preference for particular stimulus

Secondary outcome

- No significant differences between types of dementia

- Participants remained on the Somatron bed for 15 mins which was unusual; these patients tend to move frequently and wander, agitated, and remaining on the bed without physical or verbal prompts to do so means it was conducive to some quieting / relaxing experience.
- Severely regressed persons with dementia do not seem to prefer vibrotactile stimulation (Somatron) to auditory stimulation or silence. However, issues with sample size and results possibly an artefact of testing conditions and stimuli used confound interpretation. Participants may not have had the ability to make choices, were reluctant to make decisions or not motivated to make the choices due to the severity of dementia.
- Participants also had routine changes: not allowed to sleep in the afternoon, which may have affected their wellbeing.
- Possibly the unfamiliar environment (music and other sound vibrating through the bed) may have been strange for participants.

Table 2.

Clements-Cortes, Ahonen et al., 2016. Short-term effects of rhythmic sensory stimulation in Alzheimer's disease: An exploratory pilot study.

Country, Setting, Language
Canada, healthcare facility with in- and out-patients, English

Study design / research experiment description
(sham, blinding, method of allocation, methodological challenges mentioned by researchers, etc.)

- Randomised crossover trial (pilot study)
    - 6 sessions low frequency intervention
    - 6 sessions DVD / visual stimulation
    - 2-day washout period between crossover
- Method of randomisation not described
- No allocation concealment or blinding procedure mentioned
- Sessions 2x week for 6 weeks, each session lasted 35-40 minutes and included either 30 min visual stimulation (DVD) or 30 min 40Hz stimulation

Methodological challenges

- Method of accurately measuring small changes in cognition (neuroimaging proposed as supplement to questionnaires)
- Frequency of sessions (ideally 3 or more sessions LF per week rather than only 2)

Objectives / hypothesis / goals

- Repeated somatosensory sessions of 40Hz would drive an increase in rhythmic oscillatory coherence at the 40 Hz gamma level and consequently contribute to improved cognition in patients at stages 1, 2, and 3 of Alzheimer's disease compared to repeated non-rhythmic visual stimulation sessions.
- The study was conducted to inform a larger proof of concept study followed by a clinical trial. The purpose of the study was to test whether the stimulation could improve alertness, cognition, and short-term memory.

Research sample, type of dementia (diagnosis), comorbid/ secondary diagnosis(es)

- N=18 participants (6 mild, 6 moderate, 6 severe) with Alzheimer's disease
- 2 participants withdrew during the study (DVD group) and were not included in data analysis
- Comorbidities/secondary diagnoses not reported

Participant characteristics
(age, gender, education, profession)

- Participants from a healthcare facility
    - n=10 males
    - n=8 females
    - 59-93 years
- Demographics (education, profession) not reported

Tactile low frequency intervention characteristics
(type of vibration [sound, mechanical], frequency (Hz), device, pulsation / cycle duration, amplitude (dB), duration (time), absence/presence of music listening, absence/presence of therapeutic relationship)

- 40Hz, 30 min, delivered with Next Wave Physioacoustic Chair
- Amplitude cycle (silence 0 dB to n > 0 dB) duration: 2.3 secs
- Directional movement pattern repeated throughout:

  - 2 min constant sound from all speakers
  - 2 min progressive movement from head to legs
  - 2 min progressive movement from legs to head.
- Max amplitude:
  - Neck area 102.4-103.2 dBc
  - Back area: 104.7-105.4 dBc
  - Thigh area: 109.1-109.6 dBc
  - Leg area: 104.9-105.3 dBc.
- Frequencies:
  - Scanning from 39.96-40.06 Hz.
- No music listening recorded. Therapeutic process not reported, although intervention was delivered by trained music therapist

Music choice
Not recorded

Participant/client or therapist chosen music listening
N/A

Outcome measures
Primary outcome measures
- Saint Louis University Mental Status (SLUMS) (total 13 measurement points, pre-study and post- each session)

Secondary outcomes measures
- Observed Emotion Rating Scale (pre- and post-session)
- Researcher Observation (body language, mood, memory, alertness, cognition; recorded during each session)

- Each subject participated in individual testing for 15 minutes, three times a week for six weeks (total 18 testing sessions). The initial two sessions were testing sessions and not included in the analysis.
- The measurement conditions, internal reliability etc. as well as who conducted the measurements is not provided
- Neural oscillation coherence is hypothesised however no measurements of brain activity e.g., EEG were conducted

Outcomes
Primary outcome
- Positive SLUMS regression slopes (0.581, increase of 0.5 per session) in LF intervention group
- 0 or negative regression slope in DVD control group
- Significant difference between LF and DVD treatment regression slopes – LF stimulates cognition, while the DVD proved to rather induce a quieter and sleepy affect. The authors reported that this sedative effect of the DVD nevertheless had no effect on anxiety / agitation.

Secondary outcomes
- *Observed emotion rating scale:* No apparent significant contribution to affect from pre- to post-session; as a result, no statistical analysis was performed
- *Four themes for LF group:*
  - Increased awareness of surroundings
  - increased interaction
  - stimulation of discussion/storytelling
  - increased alertness
- *Four themes for DVD group:*

- Boredom
- Increased agitation/anxiety
- quiet
- sleep

The outcomes of the qualitative thematic analysis support the outcomes from the quantitative part.

Table 3.

Clements-Cortes et al., 2017a Can Rhythmic Sensory Stimulation decrease cognitive decline in Alzheimer's Disease? A clinical case study

Country, Setting, Language
Canada, Long-term facility and home, English

Study design
- Case study (mixed methods)
- Follow up (after 3 years) reported
- no blinding
- limitations reported: limitations of statistical conclusions, potential subjectivity of the therapist or their observations and assessments, interview questions not standardized

Objectives / hypothesis / goals
- Neural oscillation can become dysregulated resulting in medical conditions. Intra-brain connectivity is also associated with 40 Hz gamma wave oscillation. External rhythmic stimulus can regulate the dysregulated circuits and connectivity. Sound vibration can increase 40 Hz activity and brain connectivity.

Research sample, type of dementia (diagnosis), comorbid/secondary diagnosis(es)
- N=1 participant
- Alzheimer's disease (MMSE Score 22/30, probably AD with mild impairment)
- Comorbidities not reported

Participant characteristics
(age, gender, education, profession)
- 92 years
- Female
- MMSE Subscores: 6/10 orientation, 3/4 on "world" backwards, 1/3 on delayed recall
- Visuospatial skills on clock drawing and intersecting pentagons were intact, but Necker Cube impaired
- CERAD learning and recall, 2, 4 and 5 words learned on the first 3 trials; 1 word recalled after a delay and 17/20 words recognised
- 18/30 on Boston Naming Test
- Verbal Fluency was average for letters C (n=14), F (n=8), and L (n=11).
- Semantic fluency for animals impaired at 10
- Digit span was 6 forwards and 4 backwards
- Trails A completed in 94 seconds. Trails B sample incomplete.
- Some preservative behaviour issues on drawing alternating Ms and Ns as well as ramparts.
- She was on a stable dose of Donepezil.
- No further demographics or patient history reported

Tactile low frequency intervention characteristics
- Low frequency sound vibration delivered over 12 sessions over 4 weeks (3 per week) using the Next Wave Physioacoustic Chair
- Daily use of commercial device Sound Oasis (VTS1000) for 30-60 mins per day, 5 times per week using Energize programme (mixture of vibration and music); 60 mins of stimulation is 20 mins of 40 Hz stimulation

- 4 sec cycle amplitude, sound moving progressively from legs to head and back again; peak amplitude 104-109 dBc. 12 45-min sessions; relaxing music during sessions 7-12 (ambient sound), intervention delivered by a certified music therapist

Music choice

- Relaxing ambient music (not specified); applied from session 7 – 12 due to boredom

Participant/client or therapist chosen music listening

- Pre-programmed music in Energize programme with Sound Oasis

Outcome measures

Primary outcome measures

- Saint Louis University Mental Status (SLUMS) cognitive assessment pre and post treatment
- MMSE also reported but not collected as part of the study

Secondary outcomes measures

- 11 questions about family (to assess long-term memory)
- Qualitative observations and reflective notes recorded by the music therapist (related to e.g. client's state, focus, confusion)
- Interviews with husband (end of each treatment week)

Outcomes

Primary outcome

- Mean SLUMS score pre-treatment: 17.67
- Mean SLUMS score post-treatment: 18.17
    - Upward linear trend in scores per treatment
        - Post-test SLUMS and post-test 11 questions data analysed to determine effect of treatment using linear regression.
        - Scores modelled as linear functions of number of treatments
        - Estimated slopes and standard errors given by 0.476(0.197) and 0.567(0.122) respectively.
        - P-values are 0.036 and 0.002 respectively.
        - Approximate effect of 0.5 of a scale point per treatment.
    - MMSE was also 22/30 post-onset and at end of 3 years daily LF (annual decline of 3.3 is typical)

Secondary outcomes

- 11 questions
    - upward linear trend in scores per treatment; 13.2 after initial 12 sessions; 10 after 3 years
- Interviews with husband
    - Little change in short-term memory initially
    - mental clarity fluctuated
    - math skills remained strong
    - crosswords becoming more difficult
    - after the last session, reported slight improvement in short term memory and sleep, generally content, some irritability
    - after 3 years, frustration has subsided with the daily LF treatments
    - can still remember children's names, but not grand- or great grandchildren.

Table 4.

Clements-Cortes et al., 2017b The potential of rhythmic sensory stimulation treatments for persons with Alzheimer's disease.

Country, Setting, Language See Clements-Cortes, Ahonen et al. 2016

Study design

- Qualitative data reported from dataset of Clements-Cortes, Ahonen et al. 2016 – a crossover trial 6 sessions vibroacoustic stimulation followed by 6 sessions of DVD
- Qualitative research questions and objectives are missing (only the quantitative part reports a hypothesis and objectives). There is a lot of information reported about the quantitative part e.g. description of the quantitative outcome measures
- The qualitative data came from thematic analysis of research observation notes. The study also includes a case vignette about "Louis" offering a description about the participant's experiences (the data are rather descriptive due to the lack of ability to self-reflect due to the disease)
- *Challenges:*
    - 3 rather than 2 sessions would be better (feasibility issue in driving to the treatment facility)

Objectives / hypothesis / goals

- Qualitative examination of important themes and interactions – to further clinical understanding of how RSS improved cognition – from a previously published quantitative report with N=18.

Research sample, type of dementia (diagnosis), comorbid/secondary diagnosis(es)

- Moderate Alzheimer's Disease

Participant characteristics (age, gender, education, profession)

- 68-year-old male

Tactile low frequency intervention characteristics

- See Clements-Cortes, Ahonen et al. 2016

Music choice

- See Clements-Cortes, Ahonen et al. 2016

Participant/client or therapist chosen music listening

- See Clements-Cortes, Ahonen et al. 2016

Outcome measures

Primary outcome measures

- SLUMS (see Clements-Cortes, Ahonen et al. 2016)

Secondary outcomes measures

- Case vignette and qualitative themes from vibroacoustic sessions and DVD sessions, analysed by thematic analysis

Outcomes

Primary outcome

- *See* Clements-Cortes, Ahonen et al. 2016

Secondary outcomes

- Mild AD participants

- Vibroacoustic: More alert, recounting interesting stories from the past, awareness of surroundings, commenting on the treatment (relaxing, interesting).
- DVD: boredom, increased anxiety, restlessness, tired, confusion

- Moderate AD participants:
  - Vibroacoustic: Increased arousal (verbal output), energy and physical action, increased voice volume, singing, general alertness
  - DVD: confusion, boredom, sleepiness, anxiety and restlessness, less talkative
- Severe AD participants:
  - Less engagement in both treatments, less coherent verbal communication, several with severe cognitive impairment showed little difference between two treatments
  - During Vibroacoustics; more evidence of interactive verbal communication, alertness, relaxing behaviour, greater awareness of context.
  - DVD: greater signs of boredom, tiredness, agitation/restlessness, outbursts

Table 5.

Heesterbeek et al., 2019a
Feasibility of Three Novel Forms of Passive Exercise in a Multisensory Environment in Vulnerable Institutionalized Older Adults with Dementia.

Country, Setting, Language
The Netherlands, nursing homes, English

Study design / research experiment description
(sham, blinding, method of allocation, methodological challenges mentioned by researchers, etc.)

- Single blind randomised controlled trial with three intervention arms and 1:1:1:1 allocation ration
- Three passive exercise types are compared
  - Whole body vibration (WBV): 6 weeks, 4 sessions/week, 12 mins
  - Therapeutic motion simulation (MSim): 6 weeks, 4 sessions/week, 4 mins,
  - combination of TMSim & WBV: 6 weeks, 4 sessions/week, 12 mins,
  - Control group: regular care
- Stratified for gender, MMSE score, age and nursing home (1:1:1:1 allocation ratio, 30 residents per group)
- Random number generator was used for randomisation
- Challenges:
  - Severity of disease: usually persons with severe dementia are excluded from studies and dropouts reported dizziness even when the devices were not running
  - Threat of overstimulation for persons with dementia
  - Interventions run by a research assistant, not nursing home staff: feasibility of application outside study setting is not known
  - Potentially fewer frail participants due to inclusion bias as a result of protective legal representatives for potential participants

Objectives / hypothesis / goals

- Increasing physical activity in patients with dementia can reduce pathology severity and disease progression. Determine the feasibility of three different forms of passive exercise (Therapeutic Motion Simulation – TMSim), Whole body vibration (WBV), and their combination TMSim & WBV) in a multisensory environment with inactive care home residents with dementia

Research sample, type of dementia (diagnosis), comorbid/ secondary diagnosis(es)

- N=120 residents from closed wards of eight nursing homes
- Alzheimer's disease (most common), vascular dementia, Lewy Body Dementia, frontotemporal dementia, combination, other/unknown dementia,
- Specific comorbidities not reported, but prevalence of comorbidities:
  - TMSim : 2.8(1.8)
  - WBV: 3.6(2.0)
  - TMSIM + WBV: 3.4(2.0)
  - Control: 3.7(2.7)

Participant characteristics
(age, gender, education, profession)

- Age range:
  - TMSim : 69-95
  - WBV: 75-96
  - TMSIM + WBV: 69-103

  - Control: 70-99
- % Females
  - TMSim : 70
  - WBV: 63.3
  - TMSIM + WBV: 66.7
  - Control: 66.7
- Further demographics not provided

Tactile low frequency intervention characteristics

(type of vibration [sound, mechanical], frequency (Hz), device, pulsation / cycle duration, amplitude (dB), duration (time), absence/presence of music listening, absence/presence of therapeutic relationship)

- Mechanical vibration
- WBV: 30 Hz, amplitude 1-2 mm, 4 mins, stationary motorcycle with idling engine is shown on screen, matching sounds
- TMSim: 3 short, real life movies of multiple activities, matching music and sounds, platform moves synchronously (visual, auditory, tactile and proprioceptive stimuli)
- TMSim + WBV: Alternating between TMSim (4 min) and WBV (2 min) for 12 mins total;
- Commercially available motion simulation device: Pactive Motion;
- Music and sounds shown on screen i.e. not participant chosen
- No therapeutic relationship

Music choice

- Matching music and sounds played with the movies in TMSim and WBV

Participant/client or therapist chosen music listening

- TMSim chosen by participant (categories included e.g., horse riding, walking, dancing, nature e.g. seaside) or proxy if needed

---

Outcome measures

Primary outcome measures

- Feasibility measured by:
  - *Attendance*: percentage of the offered sessions that were actually attended
  - *Compliance*: percentage of attended sessions that were completed according to protocol
  - *Experience scores*: scales between 0 (very unpleasant) and 10 (very pleasant), obtained for each completed session
  - *Adverse events and dropout rates*: a distinction made between dropouts related to the intervention (e.g. unwillingness to participate after a number of sessions or dropouts related to adverse events) and dropouts not related to the intervention (e.g. sickness and death)
- "An intervention was considered as feasible when attendance was high (|75%), interventions were completed according to protocol (>90%), experience scores indicated at least pleasant experiences (≥6.5) for the participants, drop-out rates related to the intervention were low (<20%), and no serious adverse events occurred" (p.4).

Outcomes

Primary outcome

- *Attendance*: 20.4±6.9 sessions offered, 16.6±7.8 were attended
  - Reasons for missed sessions:
    - Motivational problems (40%)
    - bedday (23%),

- sick (9.1%)
- not present at ward (7.9%)
- visitors (5.5%)
- delusions (3.9%)
- physical discomfort (3.3%)
- tiredness (3.3%)
- aggressive behavior (2.1%)
- discommended by staff (1.2%)
- disoriented (0.3%)
- broken wheelchair (0.3%)

- *Compliance*:
  - 65% of participants attended 24 sessions they were offered
  - 12% were offered 23 sessions
  - 15% were offered 22
  - 6% were offered 21 sessions
  - 1.5% were offered 20 sessions.
  - 90 assigned to an intervention group, 68 attended at least 12 (50%) of the scheduled sessions. Cases of non-compliance were caused by participants wanting to leave before the session was finished
- *Mean adherence rates;*
  - TMSim + WBV: 85%
  - TMSim and WBV: 90%;
  - 99.3% compliance of attended sessions / completed per protocol
- *Experience scores:* 52 of 90 participants were able to judge the sessions and indicated the sessions were pleasant (WBV: 6.9; TMSim + WBV: 7.3). Proxy scores tended to be better
- Most watched category: walking; least watched: extreme sports
  - Large variation in preferred categories in both TMSim and TMSim+WBV groups
- *Adverse events and dropout rates*: some motion sickness (n=4) (n=2 reported motion sickness also when device was turned off)
- *The intervention can be easily extended or shortened depending on the needs of the individual;*

** QoL and ADL were mentioned in the protocol for the study but were not reported in the full-length study*

Table 6.

Kim & Lee, 2018
The effects of whole body vibration exercise intervention on electroencephalogram activation and cognitive function in women with senile dementia

Country, Setting, Language
South Korea, community-dwelling participants, English

Study design / research experiment description
- Quasi-experimental study
- Control group: probably no intervention, but it is not specified in the paper
- 5 sessions per week for 8 weeks
- randomization not specified
- blinding not specified
- method of allocation not specified

Objectives / hypothesis / goals
- The study provided a WBV intervention to women with senile dementia and examined the effects of it on EEG activation and cognitive function.
- Exercise therapies are known to activate brain metabolism, increase cerebral blood flow, stimulate neurotransmitter secretion, and enhance functioning and thereby increase quality of life in persons with dementia
- EEG activation shows the functional state of the brain but patients with dementia indicate lower EEG activation than those without dementia.
- The purpose is to provide foundational material to improve quality of life in patients with senile dementia by developing an effective interventional exercise program

Research sample, type of dementia (diagnosis), co-morbid/ secondary diagnosis(es)
- Participants aged 65 or older suspected of having mild dementia (23 points or lower on MMSE-K)
- community-dwelling participants
- comorbidities not mentioned
- type of dementia not specified

Participant characteristics
- Exercise group (9 participants): 79.22(4.02) years; 156.48(2.08) cm; 57.03(2.71) kg;
- Control group (9 participants): 81.44(3.75) years; 156.84(2.81) cm; 58.87(2.72) kg
- All female participants
- Education and profession not specified

Tactile low frequency intervention characteristics
- Mechanical vibration delivered using whole body vibration exercises (VM-10, Korea)
- Exercise included 5 sets of standing, squat and sumo squat positions lasting 2 min and 1 min rest between positions (total 15 mins)
- Frequency: 20Hz, increased gradually by 5 Hz every 2 weeks (up to 35 Hz).
- Duration: 5 times per week for 8 weeks (40 sessions, 40 mins; 3*2 mins with 5 reps = 30 + 1 min rest between positions, 10 mins rest total) with light whole-body stretching as warm-up and –down
- Amplitude not mentioned
- No music listening

- No therapeutic relationship

Music choice

- N/A

Participant/client or therapist chosen music listening

- N/A

Outcome measures

Measurements before and after the whole intervention programme for both groups (pretest-posttest design)

Primary outcome measure

- EEG activation (measured by LXE3208, LAXHA Inc., Daejeon, Korea)

Secondary outcomes measures

- Mini-Mental State Examination-Korean version (MMSE-K)

Outcomes

Primary outcome

- Significant improvement in EEG activation after WBV exercise intervention, measured using EEG (p=0.001 group x time comparison for all 8 points; WBV elevates EEG activation positively in women with senile dementia

Secondary outcome

- Significant improvement in MMSE-K: Pretest-posttest difference: EG = 20.889 x 23.556; CG =21.333 x 20.899; group x time difference p=0.001

Table 7.

Lam et al., 2018
Effects of adding whole-body vibration to routine day activity program on physical functioning in elderly with mild or moderate dementia: A randomized controlled trial

Country, Setting, Language
Hong Kong, day-care centre, English

Study design / research experiment description
(sham, blinding, method of allocation, methodological challenges mentioned by researchers, etc.)

- Single-blind, randomised controlled trial with two arms and 1:1 allocation ration (WBV + routine activity programme vs routine activity programme only)
    - Activity programme: active limb mobilisation exercises, lower limb strengthening exercises, walking and balance exercises, various social and cognitive activities
    - WBV intervention (described below)
    - Two sessions per week for 9 weeks, weeks 1-3: 4 mins vibration (30 secs with 1-2 min break); weeks 4-9: 6 mins with 1-2 mins break between 45 sec vibration)
- Online randomisation program (not specified) used by independent researcher not involved in assessment/training programme
- Outcome assessors blinded
- Method of allocation concealment not mentioned

Objectives / hypothesis / goals

- To evaluate the effects of adding WBV to a usual activity program provided at day-care centres on physical functioning among elderly adults with mild or moderate dementia

Research sample, type of dementia (diagnosis), comorbid/ secondary diagnosis(es)

- 54 Community-dwelling individuals attending day-care, mild or moderate dementia
- *WBV group:* CMMSE* score 13.6 (4.7)
- *Control group:* CMMSE score 15.6 (4.5)

Comorbid conditions per person (hypertension, diabetes, high cholesterol, depression, history of stroke):

- *WBV group* 3.6 (1.3) per person,
- *Control group* 3.3 (1.4) per person

The minimum sample size was calculated for the study

Participant characteristics
(age, gender, education, profession)

- N=54 Community-dwelling individuals
- *WBV group*: men/women 8/19, 79.7 (5.5) years old
- *Control group*: men/women 6/21, 79.9. (6.7) years old

Tactile low frequency intervention characteristics
(type of vibration [sound, mechanical], frequency (Hz), device, pulsation / cycle duration, amplitude (dB), duration (time), absence/presence of music listening, absence/presence of therapeutic relationship)

- Mechanical vibration (Pro 5 Power Plate)
- Vertical vibrations, frequency 30 Hz, peak-to-peak amplitude 2mm, 2 sessions per week
    - 8 bouts of vibration applied in each session; weeks 1-3 30s bouts, weeks 4-9: 45s bouts (total 4-6 min exposure per session, 1-2 min rest between bouts)

- Standing on platform, static and dynamic semi-squats (90° flexion, range of 20°-90° flexion), repeated 4 times, 8 per session

Music choice
- N/A

Participant/client or therapist chosen music listening
- N/A

Outcome measures
- Outcomes assessment was conducted 3 times; baseline, immediately after the training programme, and at 3 months after the end of the training programme (follow-up).

Primary outcome measures
- Functional mobility - Timed up-and-go (TUG) test

Secondary outcomes measures
- Balance - Berg Balance Scale (BBS) and Tinetti balance assessment
- Lower body strength - 5-time-sit-to-stand task
- Cognitive performance - Activities-specific Balance Confidence scale
- Quality of life - Cantonese version of Quality of Life in Alzheimer's disease (QOL-AD)

The attendance rate and adverse events were also recorded

Outcomes

Primary outcome
- Significant improvements in mobility and balance independent of group allocation
- TUG improved at 3-month follow-up but not at post-training assessment
  - WBV: ES: 0.073, p=0.021*
  - Control: ES: 0.006, p=0.707

Secondary outcomes
- Berg Balance Scale (BBS) and Tinetti significantly higher post-training
  - BBS: WBV → ES 0.124, *p*=0.001*; Control → ES 0.011, *p*=0.571
  - Tinetti: WBV → 0.033, *p*=0.187; Control → ES: 0.017; *p*=0.382
  - Tinetti Balance Subscale_ WBV: ES 0.064, *p*=0.041*
- QoL-AD reduced significantly post-training and further at 3-month follow-up
  - WBV → ES=0.018, *p*=<0.001*, Control → ES=0.029, *p*=0.221
- The attendance rate was high (86%) and adverse events rate low (two of 27 participants reported mild knee pain).

* Chinese Mini Mental State Examination

Table 8.

Mercado & Mercado, 2006
A program using environmental manipulation, music therapy activities, and the Somatron Vibroacoustic chair to reduce agitation behaviors or nursing home residents with psychiatric disorders

Country, Setting, Language
USA, psychiatric nursing facility, English

Study design / research experiment description
(sham, blinding, method of allocation, methodological challenges mentioned by researchers, etc.)

- Single-case studies (observational-descriptive)
- Three-step programme:
    - **Intervention 1:** develop calm atmosphere in each of the 5 facility units (noise reduction, specially chosen music at specific times of day; Morning music 9am-12pm = rhythmic and stimulating "Moonlight sonata", "Winter" from Vivaldi's *The Four Seasons*; Afternoon music 12-5pm: flowing, less rhythmical, soothing (no examples given). Staff instructed to lower volume of conversations / reduce amount of uncontrolled radio / television
        - All unnecessary intercom announcements discoursed. Speakers installed on all units and controlled background music applied in common living areas (instrumental and music with lyrics used)
    - **Intervention 2**: increased organised activities / leisure activities (was not implemented due to structural changes) in Unit 3 (aggressive) → *not implemented due to administrative decisions to disperse Unit 3 residents among others*
    - **Intervention 3:** music therapy offered to residents showing no change from Intervention 1 (active or receptive structured sessions, vibroacoustic sessions, or combination). Individualised interventions – singing familiar songs, playing rhythm instruments, movement activities, listening to music. Vibroacoustic therapy was only used in Intervention 3. Case studies are reported from this intervention.
- *Challenges:*
    - **Participant 1**: would not agree to go to Somatron room (received no vibroacoustic stimulation)
    - **Participant 2**: enjoyed sessions; refused to attend after she had been moved to another unit before session 7 and was agitated (active music therapy intervention lasted for 3 months thereafter)
    - **Participant 3**: scheduling conflicts, hospitalisation, family visits, refusal to participate
    - Flexibility required to implement a new programme and document outcomes
        - Inconsistent sessions due to symptoms and unforeseen schedule changes, facility priorities changed and admin decisions altered the programme

Objectives / hypothesis / goals

- To document the effects of a music therapy program consisting of environmental interventions (intervention 1), background music (intervention 2), and individualised receptive and active music therapy sessions, and vibroacoustic sessions using the Somatron to decrease agitation behaviours of nursing home residents with psychiatric disorders (intervention 3)

Research sample, type of dementia (diagnosis), comorbid/ secondary diagnosis(es)

- Interventions 1-2:

- n=135 residents of 5 Units of a nursing facility operated by a state mental health institution. Admission required having a psychiatric diagnosis and a physical or mental disability that prevented functioning in the community without substantial assistance
- Mean age = 68 years
- 1 closed unit due to wandering behaviours / elopement risks (mixed ward). Doors locked continuously; leaving only possible with an escort; 1 unit classified as aggressive/combative (mixed ward); remaining wards male or female.
- Primary diagnoses: dementia, schizophrenia; further diagnoses were bipolar disorders, personality disorders, drug-related disorders

- Intervention 2:
  - Not implemented.
- Intervention 3:
  - Participant 1:
    - Mental disorder unspecified
    - Status Post Head Trauma with surgery removal of subdural hematoma
    - Dementia, unspecified;
    - depression
  - Participant 2:
    - Schizophrenia, chronic undifferentiated type;
    - Dementia, unspecified
  - Participant 3:
    - Dementia secondary to Brain Atrophy
    - Anxiety disorder

Participant characteristics (age, gender, education, profession)

- P1: 45-year old caucasian female
- P2: Caucasian female
- P3: female
- No further demographic details or anamneses of patients provided

Tactile low frequency intervention characteristics
(type of vibration [sound, mechanical], frequency (Hz), device, pulsation / cycle duration)

- Device: Somatron Clinical Recliner: EZ Access Model (full body "Second Diaphragm" speakers, head speakers, vinyl upholstery, motor assisted reclining mechanism for transfer, armrest lowered, seat height 4" higher)
- Sound vibration
- Bi-weekly 30 min sessions
- Sessions included music listening
- Stimulation characteristics (Hz etc.) not described

Music choice

- Options included classical e.g. "Andante" from Piano Concerto no. 21 Mozart and modern e.g. Chakra Suite by Steven Halpern

Participant/client or therapist chosen music listening

- Therapist offered options, participants indicated their preferences (when possible)

Outcome measures
Primary outcome

- **Stage 1:** Observation of the units

  - Baseline data collected (3 months) to inform which interventions to apply
    - Self-inflicted injuries and assaults and their timeframe (8:00-17:00, after 17:00) from accident and incident reports
    - Medication patterns
    - Documentation of staff absences
- **Stage 2:**
  - Activity therapy staff rescheduled to enable activities in the evening.
  - Identification of nursing staff who would conduct leisure activities with the residents (Goal: provide activities on Unit 3 until 8:30pm) – *not realised due to administrative issues*
- **Stage 3:**
  - Identify residents who did not show changes in agitation behaviour after Interventions 1 & 2
  - Subjective observation from unit staff, referral for individual music therapy (active/receptive) sessions according to resident's needs and preferences
  - Observation of these residents determined the specific behaviours to be tracked and the corresponding baseline data
    - Participant 1: spreading legs, slinging body, moving head, pulling up dress
    - Participant 2: increase duration of eyes closed while on Somatron, increase coherent verbalisations
    - Participant 3: Only two sessions attended (scheduling conflicts, hospitalisation, visits, refusal to participate). No specific behaviour tracking reported
  - *Somatron Placement Matrix* was developed considering placement criteria (response to stimuli, movement capabilities, communication capabilities, cognitive skills, physical / verbal agitation), applied music therapy intervention given current state and patient's capabilities, length of Somatron session, observations (physiological changes, relaxation, increase in positive behaviours), and music considerations.

Outcomes

Primary outcomes

- **Stage 1:**
- *Observation of residents on different units*:
  - agitation during the day (repetition of words, restlessness, aggression)
    - Assaults/injuries during 3-month period:
      - Total number reduced from 43 to 8 (82%); majority occurred after 5pm
    - Observations showed potential reasons for agitation during baseline assessment
      - Reaction to environmental noise
      - Lack of meaningful activities in the evening
      - Little control over environment (boredom as a cause for stress, escalating to agitation)
      - Only selected residents had opportunity to attend evening activities (those showing aggression less likely to attend)
      - → Three-step procedure developed as a result of these observations
- *Medication patterns:*
  - 145 PRN "as needed" (baseline) - 13 (intervention) = 91% reduction
  - 45 STAT "give medication immediately" (baseline) - 29 (intervention) = 36% reduction
- *Staff absences*: Baseline 3 months: 482 calls requesting unplanned absences, reduced to 270 absences at conclusion of intervention
- **Stage 3:**

- **Participant 1:** ***Participant refused to travel to the Somatron room and therefore did not participate in vibroacoustic / somatron sessions. She received music therapy sessions 3 times weekly, 30 minutes, intervention period 3 months. "Structured" category in Somatron Placement Matrix → individual music therapy sessions 3x weekly using variety of active and receptive participation techniques***
    - 100% decrease of two behaviours (spreading legs, slinging body)
    - 71% reduction in head moving
    - 11% reduction in pulling up dress
- **Participant 2 (6 Somatron sessions); "*Guided" Category in Somatron Placement Matrix, Somatron sessions 3x weekly.*** *Participant was moved to another unit before session 7 due to renovations and only sat on Somatron for 5 mins during Session 7 and then returned to the unit. Resident experienced high anxiety, probably due to disruption from the move. She refused to travel to the Somatron room when asked. During initial observation she showed behaviours of hitting doors and windows, screaming, throwing herself on the floor. None of these behaviours occurred during any of the sessions but other behaviours were observed indicating anxiety and these were monitored during the sessions*
    - 71% increase in keeping eyes closed during Somatron
    - 100% increase in coherent verbalisations during sessions
- **Participant 3: *"Self-Determination" category of Somatron Placement Matrix; Somatron sessions 3x weekly.*** *Only two sessions attended (scheduling conflicts, hospitalisation, visits, refusal to participate). No specific behaviour tracking reported. Appeared more positive during second session; eye contact, smiling, discussing music preferences; appeared to relax evidenced by crossed legs, head rested against headrest, arms crossed over chest, remained on Somatron for entire 30 min.*

- **Somatron Placement Matrix:** The inclusion criteria needed to be more specific due to the wide range of behaviours and functioning levels of the residents resulting in the development of the *Somatron Placement Matrix.* Common characteristics in persons who benefited from Somatron alone, Somatron as part of music therapy sessions, or if Somatron was contraindicated. Diagnosis was not a factor (evident in Participant 2's change in behaviour after unit move and subsequent refusal to attend Somatron room).

Table 9. *Critical appraisal of included studies on low frequency vibration for dementia*

Case reports

	Were patient's demographic characteristics clearly described?	Was the patient's history clearly described and presented as a timeline?	Was the current clinical condition of the patient on presentation clearly described?	Were diagnostic tests or assessment methods and the results clearly described?	Was the intervention(s) or treatment procedure(s) clearly described?	Was the post-intervention clinical condition clearly described?	Were adverse events (harms) or unanticipated events identified and described?	Does the case report provide takeaway lessons?	Quality percentage
Clements-Cortes et al. 2017a	No	No	Yes	No	Yes	Yes	No	No	58.33%
Mercado & Mercado	Yes	No	No	Yes	No	No	Yes	Yes	66.66%

Qualitative research

	Is there congruity between the stated philosophical perspective and the research methodology?	Is there congruity between the research methodology and the research question or objectives?	Is there congruity between the research methodology and the methods used to collect data?	Is there congruity between the research methodology and the representation and analysis of data?	Is there congruity between the research methodology and the interpretation of results?	Is there a statement locating the researcher culturally or theoretically?	Is the influence of the researcher on the research, and vice-versa, addressed?	Are participants, and their voices, adequately represented?	Is the research ethical according to current criteria or, for recent studies, and is there evidence of ethical approval by an appropriate body?	Do the conclusions drawn in the research report flow from the analysis, or interpretation, of the data?

Clements-Cortes et al 2017b	No	No	Unclear	Unclear	Yes	No	No	Yes	Yes	Unclear	63.33%

Quasi-experimental studies

	Are the cause' and 'effects' clear?	Participants included in comparisons similar?	Participants included in any comparisons receiving similar treatment/care, other than the exposure or intervention of interest?	Was there a control group?	Multiple measurements of the outcome both pre and post the intervention/exposure?	Follow up complete and if not, were differences between groups in terms of their follow up adequately described and analyzed?	Outcomes of participants included in any comparisons measured in the same way?	Outcomes measured in a reliable way?	Appropriate statistical analysis used?	
Clair & Bernstein	Yes	Not relevant	Not relevant	Yes	Unclear	Yes	Yes	Not relevant	No	83.33%
Kim & Lee	Yes	Yes	Yes	Unclear	Yes	Yes	Yes	Yes	Yes	88%

Randomised controlled trials

	Was true randomization used for assignment of participants	Was allocation to treatment groups concealed?	Were treatment groups similar at the baseline?	Were participants blind to treatment assignment?	Were those delivering treatment blind to treatment	Were outcomes assessors blind to treatment assignment?	Were treatment groups treated identically other than the	Was follow up complete and if not, were differences between	Were participants analyzed in the groups to which they were	Were outcomes measured in the same way for treatment	Were outcomes measured in a reliable way?	Was appropriate statistical analysis used?	Was the trial design appropriate, and any deviations

	to treatment groups?				assignment?		intervention of interest?	n groups in terms of their follow up adequately described and analyzed?	randomized?	groups?			from the standard RCT design (individual randomization, parallel groups) accounted for in the conduct and analysis of the trial?	
Clements-Cortes et al 2016	Unclear	Unclear	Yes	Unclear	Unclear	Unclear	Yes	No	Unclear	Yes	Unclear	Yes	Yes	76.92%
Heesterbeek et al.	Yes	Yes	Yes	No	No	Unclear	Yes	Yes	Yes	Yes	Unclear	Yes	Yes	84%
Lam et al.	Yes	Unclear	Yes	No	No	Yes	Yes	Yes	Yes	Yes	Yes	Yes	Yes	87%
